# A single residue change only differing by an atomic group can drive imprinting to influenza

**DOI:** 10.21203/rs.3.rs-6914018/v1

**Published:** 2025-07-07

**Authors:** Jiayi Sun, Gyunghee Jo, Chloe A. Troxell, Yanbin Fu, Robert Hoezl, Huibin Lv, Hassanein H. Abozeid, Qi Wen Teo, Tossapol Pholcharee, Joshua J.C. McGrath, Siriruk Changrob, Sean A. Nelson, Atsuhiro Yasuhara, Min Huang, Nai-Ying Zheng, Jordan C. Chervin, Lei Li, Monica L. Fernández-Quintero, Johannes R. Loeffler, Alesandra J. Rodriguez, Jiachen Huang, Olivia M. Swanson, Angel Balmaseda, Guillermina Kuan, Lora Campredon, E. Kaitlynn Allen, Gabriele Neumann, Nicholas C. Wu, Yoshihiro Kawaoka, Florian Krammer, Paul G. Thomas, Aubree Gordon, Andrew B. Ward, Julianna Han, Patrick C. Wilson

**Affiliations:** 1 Drukier Institute for Children’s Health, Department of Pediatrics, Weill Cornell Medicine, New York, NY, USA; 2 Department of Integrative Structural and Computational Biology, The Scripps Research Institute, La Jolla, CA, USA; 3 Department of Biochemistry, University of Illinois Urbana-Champaign, Urbana, IL, USA; 4 Carl R. Woese Institute for Genomic Biology, University of Illinois Urbana-Champaign, Urbana, IL, USA; 5 Influenza Research Institute, Department of Pathobiological Sciences, University of Wisconsin-Madison, Madison, WI, USA; 6 Department of Epidemiology, School of Public Health, University of Michigan, Ann Arbor, MI, USA; 7 Department of Host-Microbe Interactions, St. Jude Children’s Research Hospital, Memphis, TN, USA; 8 Division of Virology, Department of Microbiology and Immunology, Institute of Medical Science, University of Tokyo, Tokyo, Japan; 9 Pandemic Preparedness, Infection and Advanced Research Center (UTOPIA), University of Tokyo, Tokyo, Japan; 10 Department of Microbiology, Icahn School of Medicine at Mount Sinai, New York, NY, USA; 11 Center for Vaccine Research and Pandemic Preparedness (C-VaRPP), Icahn School of Medicine at Mount Sinai, New York, NY, USA; 12 Department of Pathology, Molecular and Cell-Based Medicine, Icahn School of Medicine at Mount Sinai, New York, NY, USA; 13 Ignaz Semmelweis Institute, Interuniversity Institute for Infection Research, Medical University of Vienna, Vienna, Austria; 14 Department of Poultry Diseases, Faculty of Veterinary Medicine, Cairo University, Giza, Egypt; 15 Sustainable Sciences Institute, Managua, Nicaragua; 16 Laboratorio Nacional de Virología, Centro Nacional de Diagnóstico y Referencia, Ministerio de Salud, Managua, Nicaragua; 17 Centro de Salud Sócrates Flores Vivas, Ministerio de Salud, Managua, Nicaragua

## Abstract

First described as original antigenic sin (OAS), which is deleterious, or now immune imprinting, which also accounts for beneficial effects, it is clear that immune responses to viruses tend to be biased by previous exposure to similar strains^1,2^. Various non-exclusive models for the basis of imprinting include that it results from unique features of childhood immunity^3,4^; it is driven by pre-existing serum antibodies via epitope masking^5^; or it occurs as a byproduct of residual memory following viral antigenic evolution^5^. To understand the basis and impact of imprinting from influenza, we characterized the B cell responses of young children upon consecutive first infections with divergent H1N1 and H3N2 influenza viruses. Here, we show that beyond being a primary response, there are no major phenotypic differences in the B cell response of children compared to that of adults. The distinct immunoglobulin variable (IgV) gene repertoire of influenza virus hemagglutinin (HA)-reactive B cells in children, along with increased cross-reactivity to past strains in adults, suggests significant homosubtypic imprinting in adults. As most B cells induced after consecutive infections with antigenically distant H1N1 and H3N2 are strain-specific, heterosubtypic imprinting is rare. However, these successive infections resulted in up to 6% of H1/H3 cross-reactive B cells, targeting the highly conserved central stalk epitope. These B cells express antibodies that are dominantly affected by imprinting with reduced affinity, neutralization potency, and breadth of activity. Mechanistically, H3 to H1 imprinting was caused by a single amino acid change (D46N), differing by just a carboxyl versus an amide atomic group on the central stalk epitope, resulting in a detrimental shift in the specificity of most H1/H3 cross-neutralizing B cells from seven children. We conclude that imprinting by influenza is most evident at the individual epitope level, where minor molecular differences can have a significant impact and need to be accounted for in epitope-targeting vaccine designs.

The influenza virus has posed a major threat to public health for over a century, causing epidemics that infect millions each year, resulting in hundreds of thousands of deaths^6^. Pandemics that occur at irregular intervals have a much greater impact. Current influenza vaccines offer only partial protection due to the combined challenges posed by multiple factors. The most well-appreciated mechanism of viral escape from immunity is the high rate of antigenic drift driven by mutations and immune selective pressure. This escape is facilitated because the major antigenic sites are predominantly on the globular head region of HA that mediates viral attachment^7^. While immunodominant and bound by the most protective antibodies, the globular head region is also the most rapidly evolving region, allowing viral escape^8,9^. Antibodies that bind to the HA stalk region, which mediates membrane fusion^7^ and is more conserved across diverse influenza viruses^10,11^, can also be protective, but occur at a reduced frequency^10,12^. Memory B cells have typically undergone affinity maturation to refine the affinity and specificity of antibodies^13^, and are also primed to respond more rapidly and vigorously^14,15^. Thus, in the face of viral evolution of the most protective but immunodominant epitopes, biased induction of B cell memory to less protective HA epitopes of past influenza virus strains is readily observed in recently infected or vaccinated people^16,17^ or in model systems^18–20^. These imprinted responses often show evidence of B cell clones initially elicited by childhood exposure decades earlier^17,21–23^. More distantly related influenza viruses predominantly share only functionally conserved epitopes. Thus, upon first exposure to divergent viral strains, such as the 2009 H1N1 pandemic influenza virus^10,24,25^ or avian viruses^4,26,27^, memory B cells tend to be induced against conserved and protective epitopes, reflecting a positive immune imprint. In total, mutational escape due to antigenic drift is enhanced or even dependent on biases in epitope targeting coupled with the immune memory imprint. Thus, there is ample evidence that an individual’s immune history to influenza impacts and predicts their future risk of infection or protection^4,28–30^.

While the phenomenon is likely multi-factorial, understanding the predominant drivers of immune imprinting is essential to either avoid or exploit it. Imprinting may result predominantly from residual memory after the virus mutates key epitopes, or may impact precursor/product relationships, driving how epitopes are targeted in future exposures as epitopes evolve. Imprinting by pre-existing serum antibodies can competitively shift future responses through epitope masking effects or other mechanisms such as Fc receptor (FcR)-driven modulation of immunity^2,31^. However, these effects are likely more transient because serum antibody responses wane over time. While there is ample evidence for biased protection from influenza virus infection based on childhood exposures^4,17,28–30^, there is also clear evidence of imprinted immune responses in adults to severe acute respiratory syndrome coronavirus 2 (SARS-CoV-2)^2,32,33^. Thus, it remains unclear if children’s immune systems are uniquely suited to become imprinted or if the first exposure alone drives imprinting.

Herein, we address various possible origins of immune imprinting by combining LIBRA-seq (linking B cell receptor to antigen specificity by sequencing)^34^ and monoclonal antibody (mAb) characterization, including structure-function analysis. Specifically, B cells from young children were studied after primary infections with H3N2 and then H1N1 influenza viruses, or vice versa, and compared to those from adults or naïve children after H1N1 virus infection. We observed no obvious phenotypic differences in the B cell responses of children that might account for imprinting. On first exposure, children target a spectrum of epitopes on HA like adults, but with a less refined repertoire. However, the unique composition of B cell repertoires and the cross-reactivity of antibodies from adult B cells to past strains suggest that adult B cell responses are significantly influenced by immune imprinting. Approximately 5% of memory B cells in children after heterosubtypic infections were cross-group reactive. These B cells targeted the central stalk epitope of HA and exhibited functional differences due to imprinting. In children following H3N2 and then H1N1 virus infections, a deleterious shift in specificity was detected in all isolated H1/H3 cross-neutralizing B cells, predominantly resulting from a single amino acid change (D46N) at the central stalk epitope. Since aspartic acid and asparagine differ by only a carboxyl group versus an amide group, this imprinting is driven by just a differing side chain. However, this side-chain difference involves a shift in charge, and so it could have a disproportionate impact on antibody binding. We conclude that immune imprinting is unlikely to be driven by unique features of childhood immunity. Instead, it occurs mainly due to the biased targeting of shared epitopes by residual memory B cells. Further, nearly complete imprinting of a response to a key epitope can be driven by the difference of just a single atomic group between past and current influenza virus strains.

## The B cells from adults and children are similar

**Extended Data Fig. 1:**
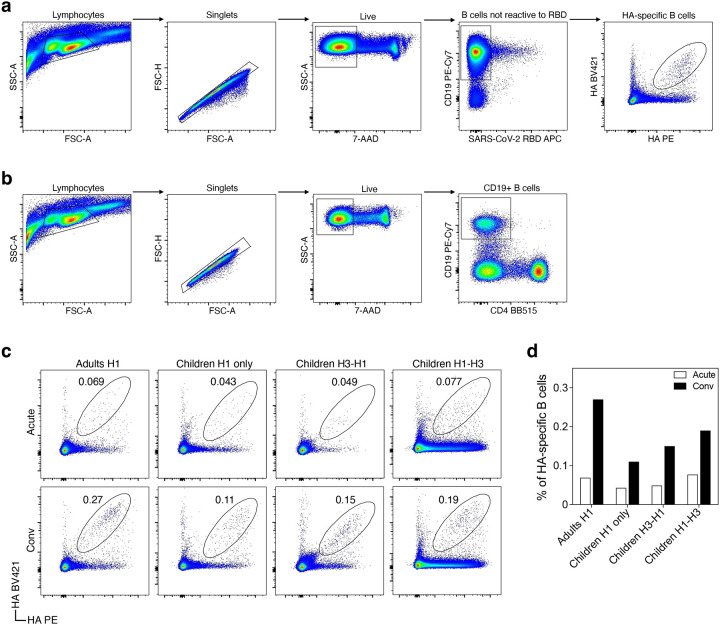
Representative flow cytometry gating for sorting CD19+ and HA-specific B cells. **a**,**b**, Representative flow cytometry plots showing gating strategy for sorting HA-specific (**a**) and CD19+ (**b**) B cells. **c**,**d**, Flow cytometry (**c**) and bar (**d**) plots showing percentages of HA-specific B cells in each group.

**Extended Data Fig. 2:**
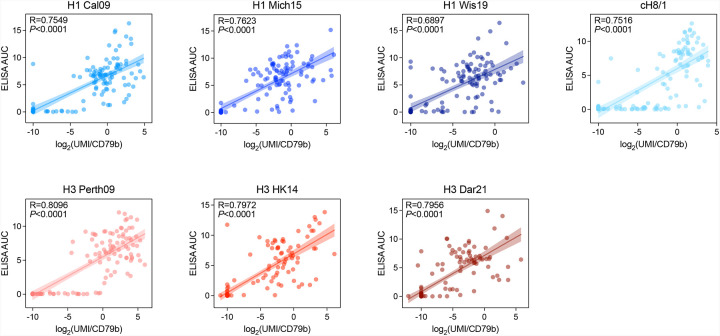
Correlation analysis on probe scores of HA-specific B cells and ELISA AUC of corresponding mAbs. Pearson correlation coefficient (R) and simple linear regression (95% confidence bands) are shown for each HA probe.

**Fig. 1:**
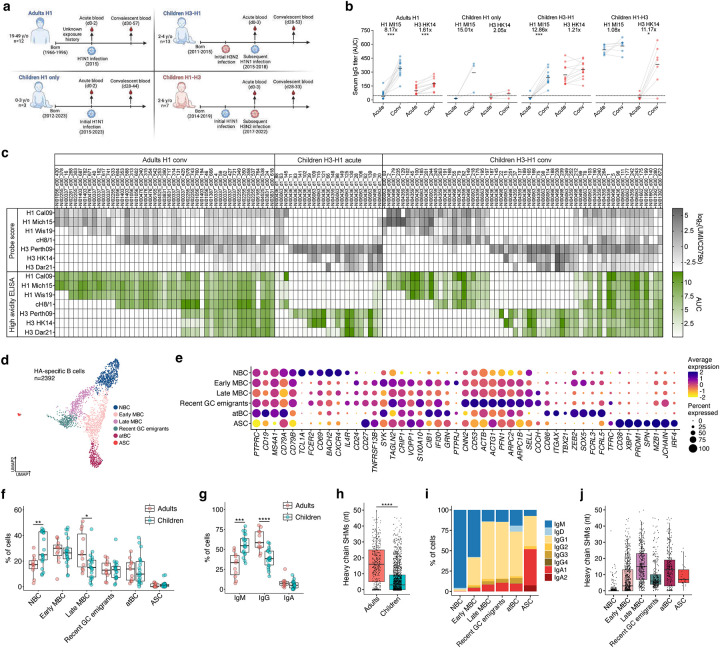
Despite a more primary response in children, B cell differentiation between adults and children are similar. **a**, Exposure history and sample collection overview for children and adults after H1N1 (blue) or H3N2 (red) virus infections. **b**, Serum IgG titers against Mich15 H1 and HK14 H3 measured by ELISA AUC, with fold changes of geometric means shown on top. Wilcoxon matched-pairs signed rank test, *P < 0.05, ***P < 0.001. **c**, Heatmap showing predicted antigen specificity (probe scores in grey) of HA-specific B cells in Adults H1 and Children H3-H1 groups, as well as validated antigen specificity (ELISA AUC in green) of corresponding mAbs. **d**, Seurat UMAP of HA-specific B cells from all subjects and time points combined. NBC, naïve B cell; MBC, memory B cell; atBC, atypical B cell; ASC, antibody-secreting cell. **e**, Expression of genes of interest for identifying annotated B cell subsets in **d**. A subset with transcriptional signatures of recent GC emigrants (upregulation of genes associated with cytoskeleton remodeling, including *ACTB*, *ACTG1*, *PFN1*, *ARPC2*) is identified. **f**,**g**, Percentages of HA-specific B cells from each B cell subset (**f**) or isotype (**g**) in adults and children. **h**, Heavy chain SHMs of HA-specific B cells in adults and children. **i**,**j**, Isotype percentages (**i**) and heavy chain SHMs (**j**) in each subset of HA-specific B cells in **d**.

To investigate the basis of immune imprinting, we collected blood samples from 2- to 6-year-old children following sequential first-time (primary) exposures to heterosubtypic influenza viruses, including 13 children with H3N2 followed by H1N1 infections (Children H3-H1), and seven children with the inverse order of infections (Children H1-H3) (Fig. 1a). For comparison, we also included three children with first-time influenza virus infections with H1N1 strains (Children H1 only) and 12 H1N1-infected adults with unknown exposure histories (Adults H1) (Fig. 1a and Supplementary Table 1). Samples were collected at the onset of symptoms (acute) and after memory B cell responses developed 4–8 weeks later (convalescent). The acute and convalescent serum IgG titers against H1 and H3 recombinant proteins confirmed the exposure history for subjects in all four groups (Fig. 1b). In line with results from a previous study^35^, cross-subtype boosts of serum antibody were observed in most children from the H3-H1 and H1-H3 groups but with significant individual variations (Fig. 1b). To dissect B cell responses to influenza infections, HA-specific B cells were bait-sorted with oligonucleotide-barcoded probes for 10X Genomics single-cell RNA sequencing (scRNA-seq), following a laboratory-adapted protocol similar to LIBRA-seq^34^ (Extended Data Fig. 1a). The probes included a panel of HAs from various seasonal influenza viruses, including those from the years of infections, along with a chimeric HA (cH8/1) with an H1 stalk and an irrelevant avian H8 head to identify HA stalk-binding B cells (Supplementary Table 2). Expectedly, all four groups showed increases in the percentages of HA-specific B cells from acute (0.043–0.077%) to convalescent (0.11–0.27%) phase, with the increase being more pronounced in adults (Extended Data Fig. 1c,d). Total CD19+ B cells from each subject were also bulk-sorted and scRNA-sequenced (Extended Data Fig. 1b). The 5’ transcriptome, B cell receptor immunoglobulin VDJ gene (BCR) repertoire, and surface feature barcode profiles were determined for 2,392 HA-specific B cells. To validate the antigen specificity of HA-specific B cells predicted by the LIBRA-seq probe scores, we synthesized variable gene cDNAs, expressed 150 recombinant mAbs from HA-specific B cells of various specificities, and tested their binding to HA probes using enzyme-linked immunosorbent assay (ELISA). The LIBRA-seq probe score and mAb binding were highly correlated (0.69<R<0.81, P<0.0001), demonstrating that the probe score accurately reflects antigen specificity of HA-specific B cells (Fig. 1c and Extended Data Fig. 2). To characterize these mAbs thoroughly, we examined their antigen reactivity, neutralization capability, epitope specificity, and molecular interactions with cognate and divergent HAs.

**Extended Data Fig. 3:**
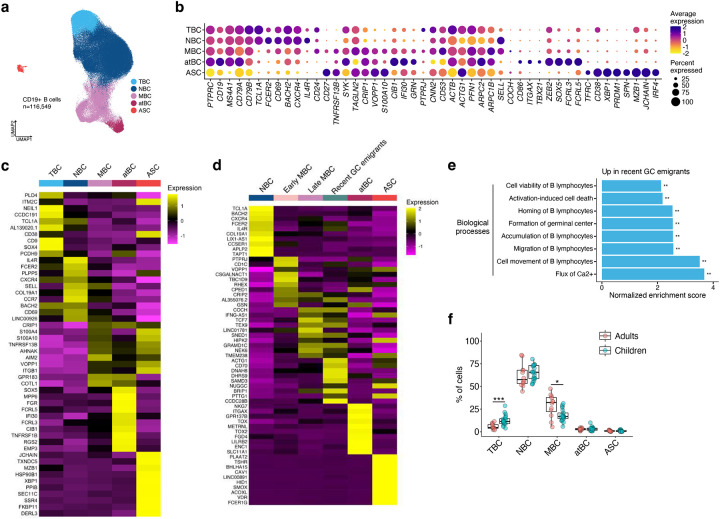
Transcriptional data of CD19+ and HA-specific B cells. **a**, Seurat UMAP of CD19+ B cells from all subjects and time points combined. TBC, transitional B cell; NBC, naïve B cell; MBC, memory B cell; atBC, atypical B cell; ASC, antibody-secreting cell. **b**, Expression of genes of interest for cells in **a**. **c**,**d**, Top 10 differentially expressed genes in each subset of CD19+ (**c**) and HA-specific (**d**) B cells. **e**, GSEA analysis showing relevant biological processes enriched in genes upregulated in recent GC emigrants from **d**. Adaptive multi-level split Monte-Carlo scheme, **P < 0.01. **f**, Percentages of CD19+ B cells from each B cell subset in adults and children.

A long-standing hypothesis is that the unique phenotypic features of immune cell subsets in young children contribute to a greater susceptibility to OAS or immune imprinting^3^. To compare B cell differentiation in children with that of adults, we separately integrated all HA-specific and CD19+ B cells for transcriptional analysis (Fig. 1d and Extended Data Fig. 3a). Based on the top 10 differentially expressed genes (DEGs), Gene Set Enrichment Analysis (GSEA) on the DEGs, and the expression of known marker genes, we identified six major B cell differentiation subsets, including: naïve B cells (NBC), transitional B cells (TBC), both early and late memory B cells (MBC), atypical B cells (atBC), antibody-secreting cells (ASC) and recent germinal center (GC) emigrants (Fig. 1e and Extended Data Fig. 3b–e). Among CD19+ B cells, there were only slightly more TBCs and fewer MBCs in children compared to adults, which is expected as children should have less immunological history (Extended Data Fig. 3f). Similarly, the overall subset distributions of HA-specific B cells were also comparable between adults and children, with more NBCs and fewer late MBCs in children (Fig. 1f). While no obvious phenotypic differences were observed between B cells of adults and young children, Ig genes from children’s HA-specific B cells were less frequently class-switched and had fewer somatic hypermutations (SHM) (Fig. 1g,h). Like the primary responses to SARS-CoV-2 mRNA vaccination^36^, recent GC emigrants were predominantly class-switched but exhibited low levels of SHM (Fig. 1i,j). Overall, these data suggest no fundamental difference in B cell phenotypes beyond a more primary response for children and more MBC re-engagement for adults.

## Robust *de novo* responses after heterosubtypic infections

**Fig. 2:**
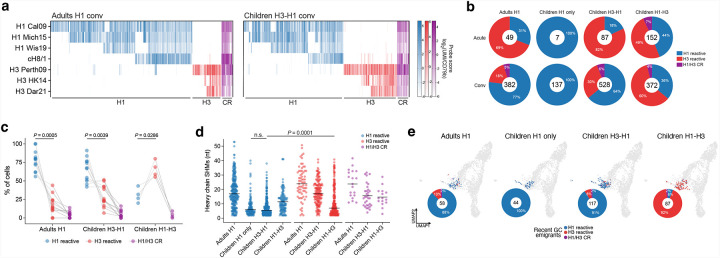
Sequential heterosubtypic infections in children induce minimal memory recall and robust *de novo* responses. **a**, Heatmaps showing predicted antigen specificity (probe scores colored by subtype reactivity) of convalescent HA-specific B cells in Adults H1 and Children H3-H1 groups. **b**, Proportions of H1 reactive (blue), H3 reactive (red), and H1/H3 cross-reactive (purple) B cells in each group at acute and convalescent time points. **c**, Same data in **b** at convalescent time point by individual subject. Only subjects with more than 10 cells are shown. Wilcoxon matched-pairs signed rank test. **d**, Heavy chain SHMs of HA-specific B cells at convalescent time point in **b**. Unpaired Mann-Whitney test. **e**, UMAPs highlighting recent GC emigrants in each group colored by subtype reactivity, with percentages shown in pie charts.

Evaluating the impact of imprinting in adults has been challenging due to the complex and unknown nature of their immune histories. To address this issue, we studied young children who had definitive first exposures through consecutive infections with both subtypes of seasonal influenza viruses (Fig. 1a). First, we used LIBRA-seq probe scores to assess the specificity of HA-specific memory B cells identified by transcriptome and the presence of BCR SHM (Fig. 2a). Consistent with flow cytometry data, we observed significant expansions in HA-specific B cells from the acute to convalescent time points (Fig. 2b and Extended Data Fig. 1c, d). At the acute time points, occurring within a few days of symptom onset during H1N1 virus infection, there was a clear bias toward H3 reactivity, which were expanded for both H3-H1 children (82%) and H1 adults (69%) (Fig. 2b). Although the adults had unknown influenza exposure histories, they may have had recent H3 exposures, as reflected by the higher H3 serum antibody levels observed at the acute time point (Fig. 1b). By convalescence from H1N1 virus infection, 4–8 weeks later, H1 reactive B cells became predominant for both H3-H1 children (70% total, including H1-specific and H1/H3 cross-reactive cells) and H1 adults (82% total) (Fig. 2b,c). Interestingly, the H1-H3 children exhibited robust expansions of H3 reactive B cells (56% total) already at the acute time point, which were further expanded by convalescence (64% total) (Fig. 2b,c). We suspect that either the H3N2 virus infection delayed symptom onset, providing more time for the expansion of H3 reactive cells between exposure and presentation, or that the response to H3N2 virus infection was more rapid, leading to earlier expansion. Supporting the notion of delayed symptom presentation, the H3-specific cells from H1-H3 children had higher BCR SHM than the H1-specific cells from H3-H1 children (Fig. 2d). In both H3-H1 and H1-H3 children, most B cells specific to the current infections had lower SHM compared to those specific to previous infections, suggesting that they were primarily *de novo* rather than back-boosted (Fig. 2d). All adult B cells showed extensively mutated IgV genes, indicating complex and repeated immune histories to H1N1 and H3N2 viruses (Fig. 2d). In children infected solely with H1N1, all HA-specific B cells were H1 reactive with few IgV SHM (Fig. 2d). For the H3-H1 and H1-H3 children, only 4 to 6% of H1/H3 cross-reactive B cells became detectable at convalescence and showed IgV genes with higher SHM than that of B cells specific to the infecting subtypes, suggesting a back-boosted origin (Fig. 2b,d). Moreover, most recent GC emigrant B cells (91–92%) in these children that participated in the current B cell response were specific to the current infecting subtypes and therefore not back-boosted (Fig. 2e). In conclusion, we find that sequential heterosubtypic infections in children result in minimal memory recall parallelled with robust *de novo* responses.

## Significant homosubtypic imprinting in adults

**Extended Data Fig. 4:**
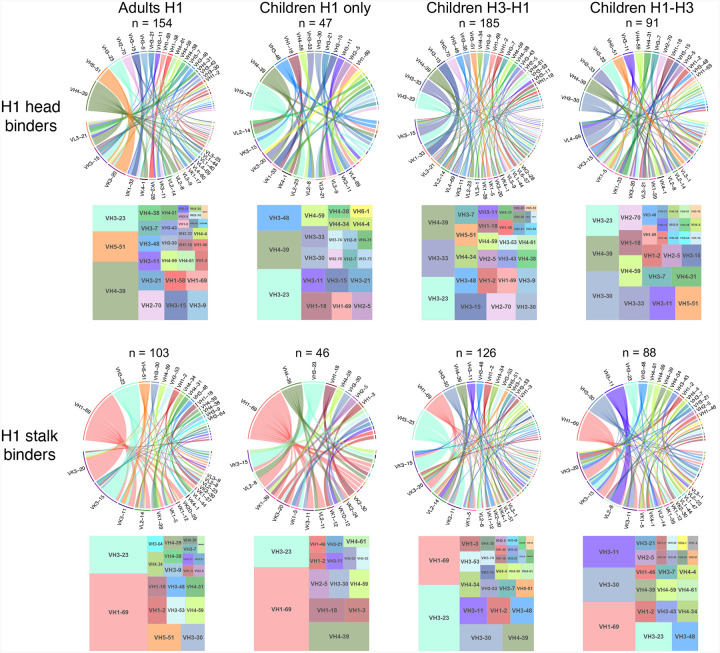
IgV gene repertoires of H1 binders from each group targeting HA head or stalk.

**Fig. 3:**
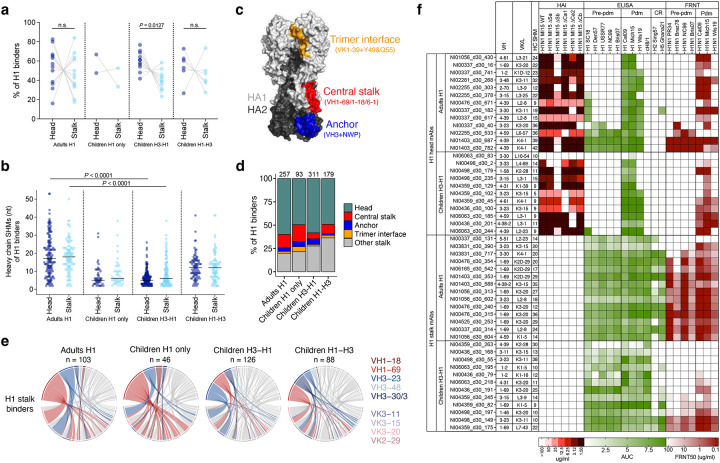
Common cross-reactivity and altered epitope targeting reveal significant homosubtypic imprinting in adults. **a**, Proportions of all H1-specific B cells (H1 binders) in each group targeting head versus stalk. Wilcoxon matched-pairs signed rank test. **b**, Heavy chain SHMs of H1 binders in **a**. Unpaired Mann-Whitney test. **c**, Surface footprints and repertoire features for indicated epitopes shown in Cal09 HA (PDB 4jtv). **d**, Proportions of H1 binders in **a** targeting each epitope. **e**, IgV heavy and light chain pairing of H1 stalk binders in **a**, with stereotypical V genes of central stalk and anchor epitopes highlighted in red and blue, respectively. **f**, HAI titer, ELISA AUC, and FRNT50 of head and stalk mAbs from subsets of H1 binders in Adults H1 and Children H3-H1 groups against indicated HAs and viruses. Results represent an average of two technical replicates from one of the two independent experiments.

We next investigated epitope and repertoire features of H1-specific B cells in adults and children for evidence of imprinting. We identified stalk-binding B cells with high probe scores to both H1 and cH8/1 probes. The *de novo* responses of H1-specific cells in H3-H1 children were significantly biased towards head-binding, consistent with the immunodominance of the head domain during a primary response (Fig. 3a). H1 adults and H1-H3 children showed large individual variations in the proportions of head- versus stalk-binding H1-specific cells (Fig. 3a). The IgV genes of H1-specific cells showed no difference in SHM between head and stalk binders within each group but were overall less mutated in children compared to adults (Fig. 3b). Across all four groups, the IgV gene repertoires of H1-specific cells showed considerable diversity. However, certain VH and VK/L pairings were enriched in multiple groups against the head (VH3–15+VK1–33; VH3–30+VL4–69; VH4–39+VL3–21/2–8/2–23) or stalk (VH1–69+VK3–20/3–15/3–11; VH3–23+VK-315/3–11) (Extended Data Fig. 4). Based on previous reports of stereotypical repertoire features and cH8/1 probe scores, we predicted the frequencies of B cells targeting common epitopes, including central stalk (VH1–69/1–18/6–1)^37^, anchor (VH3–23/3–30/3–48+NWP motif)^38^, and trimer interface (VK1–39+Y49&Q55)^39,40^. While the overall epitope distributions between groups were comparable, we observed fewer central stalk and more unknown stalk binders in children following heterosubtypic infections (Fig. 3c,d). Specifically, there were fewer H1 stalk-targeting B cells encoded by VH1–69 in H3-H1 (15%) and H1-H3 (19%) children, compared to H1 adults (34%) (Fig. 3e). The increased SHM and more stereotyped use of IgV genes suggest a more honed and memory-biased (imprinted) response from adults.

**Extended Data Fig. 5:**
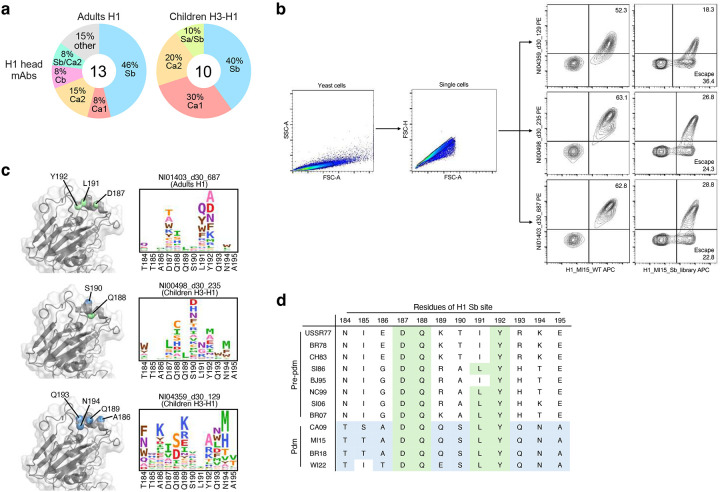
Fine epitope mapping of H1 head mAbs from adults and children. **a**, Proportions of H1 head mAbs in Fig. 3f targeting each antigenic site. **b**, Representative flow cytometry gating for sorting escape mutants from deep mutational scanning (DMS). **c**, DMS logo plots showing escape mutations against three Sb-targeting mAbs, including two narrow mAbs from children (NI04359_d30_129 and NI00498_d30_235) and one broad mAb from adults (NI01403_d30_687). The letter heights are proportional to the fractions of escape. Major contact residues targeted by each mAb are highlighted and colored based on conservation. **d**, Amino acid sequence alignment of Sb site between pre-pdm and pdm H1s, with shared residues in green and pdm-specific residues in blue.

**Extended Data Fig. 6:**
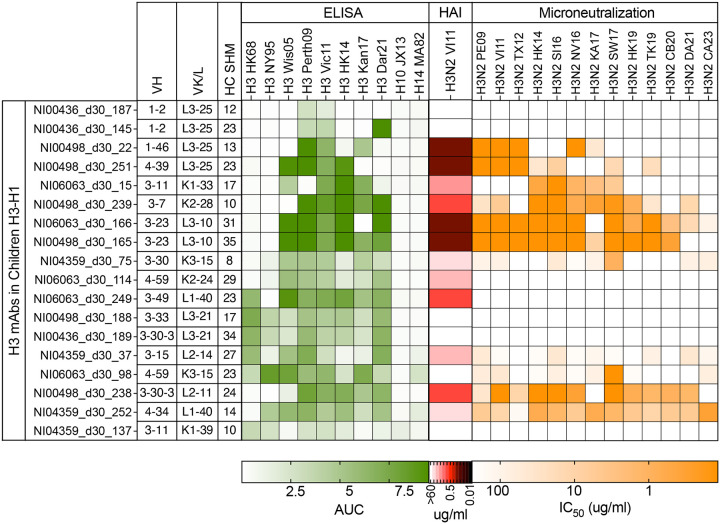
ELISA AUC, HAI titer, and microneutralization IC_50_ of H3-specific mAbs in Children H3-H1 group against indicated HAs and viruses.

Antigenic distance between influenza viruses has been repeatedly correlated with immune imprinting effects^4,17,20,28–30^, suggesting more pronounced imprinting within the same influenza virus subtype. We next evaluated homosubtypic imprinting by characterizing the cross-reactivity of H1-specific mAbs from B cells of H3-H1 children with primary H1 responses and H1 adults with complex immune histories. These antibodies exclusively bound to H1 and not H3, confirming the predictions made by LIBRA-seq probe scores (Fig. 1c). The 11 H1 head mAbs obtained from H3-H1 children showed narrow binding and neutralization solely against the closely related 2009-pandemic (pdm) H1N1 strains, indicating no influence from pre-existing memory and aligning with the low level of SHM observed (Fig. 3f). In contrast, about one third of the 13 H1 head mAbs derived from H1 adults demonstrated broad binding and neutralizing capabilities against both pdm strains and strains before the 2009-pandemic (pre-pdm). The IgV genes of these cells also exhibited a higher level of SHM, suggesting a significant origin from MBCs elicited during previous exposures to varied H1N1 strains (Fig. 3f). Fine epitope mapping analysis with viral antigenic site mutants and deep mutational scanning (DMS) mapped the contacts of two Sb-targeting mAbs from children with narrow reactivity to strain-specific epitopes, as well as one Sb-targeting adult mAb with broad reactivity to the epitopes shared by pre-pdm and pdm strains (Fig. 3f and Extended Data Fig. 5a–d). Finally, most H3-specific mAbs isolated from H3-H1 children targeted the HA head, and half of them were cross-neutralizing against multiple contemporary H3N2 strains (Extended Data Fig. 6).

**Extended Data Fig. 7:**
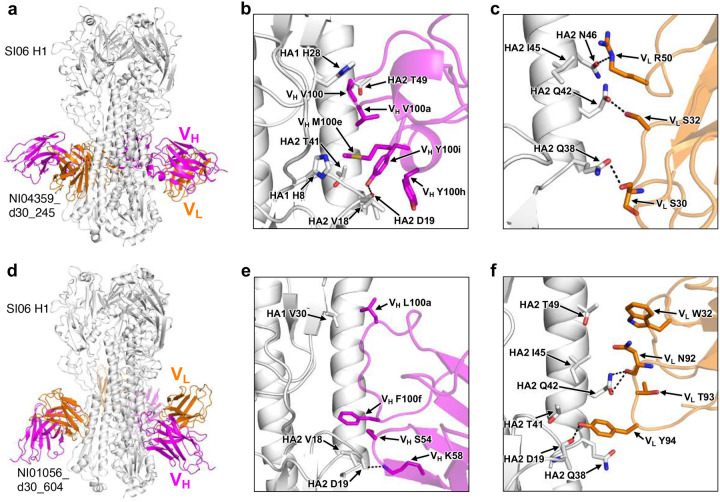
Structural analysis of two neutralizing H1 stalk mAbs using nonstereotypical IgV genes. **a**-**c**, Cryo-EM map of mAb NI04359_d30_245 from Children H3-H1 group in complex with A/Solomon Islands/3/2006 H1 (**a**) and detailed interactions of epitope residues in HA with paratope residues in heavy (**b**) or light (**c**) chain. **d**-**f**, Same data in **a**-**c** for mAb NI01056_d30_604 from Adults H1 group.

The stalk-binding mAbs specific only to H1 strains from adults and H3-H1 children were broadly reactive to H1s of both pre-pdm and pdm strains (Fig. 3f and Extended Data Fig. 7a–f). However, unlike those from adults, the H1 stalk mAbs from children were mostly not cross-reactive to H2 and H5 (Fig. 3f). The majority of the adult mAbs were broadly and potently neutralizing. In contrast, those from children exhibited less neutralization breadth and were generally poorly or non-neutralizing to the infecting A/Michigan/45/2015 (Mich15)-like H1N1 strain (Fig. 3f). While 57% (8/14) of the adult mAbs were encoded by the VH1–69 gene, only a quarter (3/12) of those from children were from this stereotypical class targeting the central stalk epitope (Fig. 3f), verifying the tendencies observed in cells with high probe scores to both H1 and cH8/1 (Fig. 3e). These results indicate that H1 stalk-reactive B cells in adults are highly selected over a lifetime of repeated exposures and imprinting, leading to robust activity against the most conserved H1 stalk epitopes. Conversely, most H1 stalk-reactive B cells elicited by the primary H1 infections in children originate from a less refined repertoire that is both more strain-specific and less potently neutralizing.

Together, the increased IgV somatic mutations, plus the cross-reactivity and more stereotyped repertoire of mAbs derived from H1N1-infected adults provide evidence of significant homosubtypic imprinting after a lifetime of repeated exposures.

## Heterosubtypic imprinting occurs in an epitope-specific manner

**Fig. 4:**
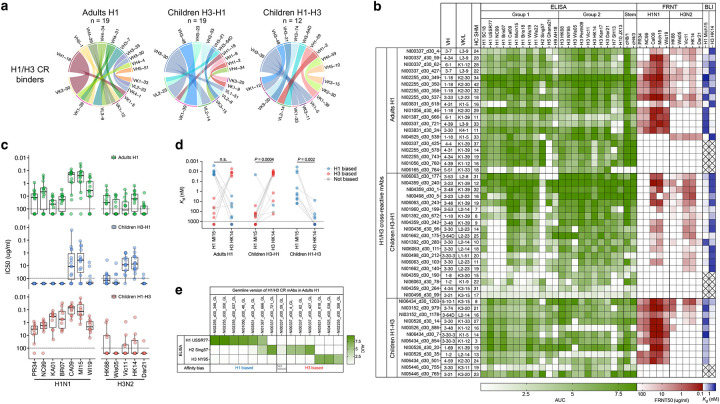
Heterosubtypic imprinting occurs in an epitope-specific manner. **a**, VH and VK/L pairing of H1/H3 cross-group reactive B cells from indicated groups. **b**, ELISA AUC and FRNT50 of mAbs, and BLI *K*_d_ of Fabs from H1/H3 cross-group reactive B cells in **a** against indicated HAs and viruses. Squares filled with ‘X’ indicate untested Fabs. Results represent an average of two technical replicates from one of the two independent experiments. **c**, Neutralization potency (IC_50_) of mAbs in **b** against indicated viruses. All values higher than 100 ug/ml are categorized as negative and replaced by 250 ug/ml. MAbs tested negative to all viruses are excluded. **d**, Equilibrium dissociation constant (*K*_d_) of Fabs from mAbs in **c** against Mich15 H1 and HK14 H3, with affinity bias indicated by color. All values higher than 1000 nM are categorized as negative and replaced by 2000 nM. Wilcoxon matched-pairs signed rank test. **e**, ELISA AUC of germline-reverted mAbs from Adults H1 group in **c** against indicated HAs.

In children with consecutive initial exposures to heterosubtypic viruses, the 4–6% H1/H3 cross-group reactive B cells represent most, if not all, of the pre-existing B cell memory to the contemporaneous strain. Therefore, we further focused our analysis of imprinting on H1/H3 cross-group reactive B cells. Notably, these B cells had IgV genes with a greater number of SHM and were overwhelmingly positive for the cH8/1 probe, indicating that most of them originated from memory B cells targeting the conserved HA-stalk epitopes (Fig. 2a,d). They exhibited highly diverse V gene locus usage for heavy (VH) and light (VK/L) chain pairings, with minimal overlap in VH genes used by adults and children (Fig. 4a). Despite this diversity, specific stereotypical V gene pairings were enriched in these B cells from H1 adults (VH1–18+VK2–30), whereas B cells from H3-H1 children utilized distinct pairings (VH3–30+VL2–14/2–23; VH3–48+VK1–39), further suggesting honing of the anti-HA repertoire in adults by the effects of repeated exposures and imprinting (Fig. 4a).

We next characterized 38 cross-group reactive mAbs, including half from seven H1 adults and the other half from seven H3-H1 children. Consistent with predictions from the LIBRA-seq probe scores, 95% (36/38) of these mAbs bound to chimeric HAs (cH8/1 and cH4/3), indicating they targeted conserved central stalk epitopes (Fig. 4b). While 68% (13/19) of mAbs from both adults and children were neutralizing to the pdm-like H1N1 viruses, the adult mAbs averaged an order-of-magnitude greater neutralization potency (Fig. 4b,c). The attenuated neutralization potency of the mAbs from children could be impacted by reduced affinity maturation, reduced selection over a lifetime, and/or a shift in specificity due to H3-imprinting. To gauge the impact of specificity shift, we closely examined the cross-reactivity of these mAbs to HAs of various influenza virus strains. Antibodies targeting the central stalk epitope are typically cross-reactive to most human H1N1 virus strains, including strains from before and after the major antigenic shift with the 2009 H1N1 pandemic, making this epitope a major target for broadly protective vaccine development efforts. As expected, most adult mAbs displayed broad reactivity to H1s and H3s from a wide range of historical and recent influenza virus strains, as well as other group 1 (H2, H5, H9) and group 2 (H7, H10) subtypes tested (Fig. 4b). Of these neutralizing cross-group mAbs from adults, 77% (10/13) were broadly-neutralizing (bnAbs) against both pre- and post-2009 pandemic strains (Fig. 4b). In contrast, while some of the cross-group mAbs from H3-H1 children bound pre-pdm strains, there was a pronounced gap in neutralization to all but the most similar H1N1 strains that have arisen after 2009. In fact, none of the 19 cross-group mAbs from seven different H3-H1 children were able to neutralize pre-pdm H1N1 strains (Fig. 4b,c), representing a gap in coverage of at least seven decades of H1N1 virus evolution.

The profound narrowing in breadth may have arisen either from the influence of memory to pre-pdm strains in adults or from the effects of H3-imprinting in H3-H1 children. To distinguish these possibilities, we characterized 12 cross-group reactive mAbs from four H1-H3 children. Notably, 83% (10/12) of these mAbs were bnAbs (Fig. 4b,c). While imprinted by H1N1 strains, these children were born after 2015 and therefore had never been exposed to pre-pdm strains. Despite lacking memory to pre-pdm strains, the bnAbs from H1-H3 children did not experience the gap, with most (9/10) neutralizing both pre-pdm and pdm H1N1 strains (Fig. 4b,c). It is also notable that, despite recent H3N2 infections, the bnAbs from H1-H3 children showed a predominance in neutralization against H1N1 strains over H3N2 strains, suggesting H1-imprinting effects (Fig. 4b,c). Further, unlike those from adults or H1-H3 children, most cross-group mAbs from H3-H1 children were not reactive to H2 strains that pose a pandemic threat, representing a further retraction of breadth (Fig. 4b,c).

**Extended Data Fig. 8:**
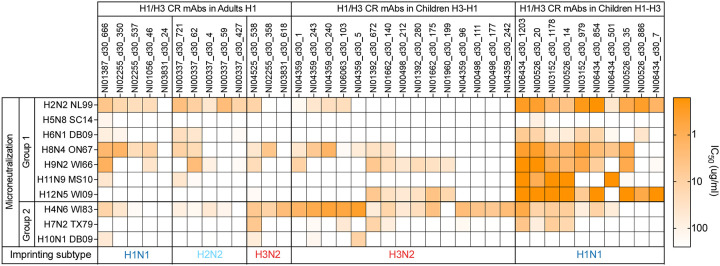
Microneutralization IC_50_ of neutralizing H1/H3 cross-group reactive mAbs from each group against indicated avian viruses.

A key predicted feature of imprinting is that the recalled MBCs will likely have a higher affinity against the imprinting strain than the infecting strain. To further evaluate the impact of cross-group imprinting, binding kinetics and affinity were measured for Fabs generated from all cross-group reactive bnAbs against Mich15 H1 and A/Hong Kong/4801/2014 (HK14) H3 using biolayer interferometry (BLI). Nearly all Fabs (13/15) from the H3-H1 children showed a higher affinity against HK14 H3 (H3 biased), whereas all those from the H1-H3 children exhibited a higher affinity against Mich15 H1 (H1 biased) (Fig. 4b,d). Finally, we analyzed the neutralization profiles of bnAbs from all three groups against avian strains with various HA subtypes. Surprisingly, H3-imprinted bnAbs from H3-H1 children potently neutralized H4N6 but not the H7N2 and H10N1 strains tested, while H1-imprinted bnAbs from H1-H3 children potently neutralized all group 1 viruses tested except the H5N8 strain (A/Sichuan/262221/2014 from clade 2.3.4.4), highlighting additional distinctions based on the imprinting exposures (Extended Data Fig. 8). Thus, consecutive heterosubtypic infections upon first exposure in children resulted in biased reactivity towards the imprinting strain for 92% (23/25) of bnAbs. Together, these findings suggest that heterosubtypic first exposure leads to imprinting on the priming strain for cross-group reactive B cells, resulting in a comprehensive shift in reactivity against the critical stalk epitopes.

Fabs from bnAbs of H1 adults showed higher affinity to HK14 H3 or Mich15 H1 in equal proportions, likely reflecting the complex immune history to influenza over a lifetime (Fig. 4b,d). The IgV genes of bnAbs from adults were highly mutated, which may have influenced the affinity bias due to affinity maturation (Fig. 2d). To confirm the imprinting subtypes of adult bnAbs, we expressed mAbs from predicted germline VDJ sequences without mutations and tested their binding to H1, H2, and H3 from strains that circulated near the birth years of these adults. Subtype specificity of germline mAbs was in line with the affinity bias of the affinity-matured bnAbs (i.e., H1-specific germline mAbs were H1-biased, while H3-specific germline mAbs were H3-biased) (Fig. 4e). Notably, a third of the germline mAbs from adults were specific to A/Singapore/1/1957 H2, suggesting these clones have been originally induced against H2N2 strains that circulated from 1957 to 1968 and then adapted to be H1-biased, H3-biased, or not biased following exposures to H1N1 and H3N2 strains over the decades (Fig. 4e).

In summary, we conclude that heterosubtypic imprinting presents in an epitope-specific manner. Furthermore, imprinting can cause significant shifts in epitope targeting or binding breadth, affecting most antibodies that cross-react with both the contemporaneous and imprinting strains.

## A single residue substitution drives H3-H1 imprinting

**Extended Data Fig. 9:**
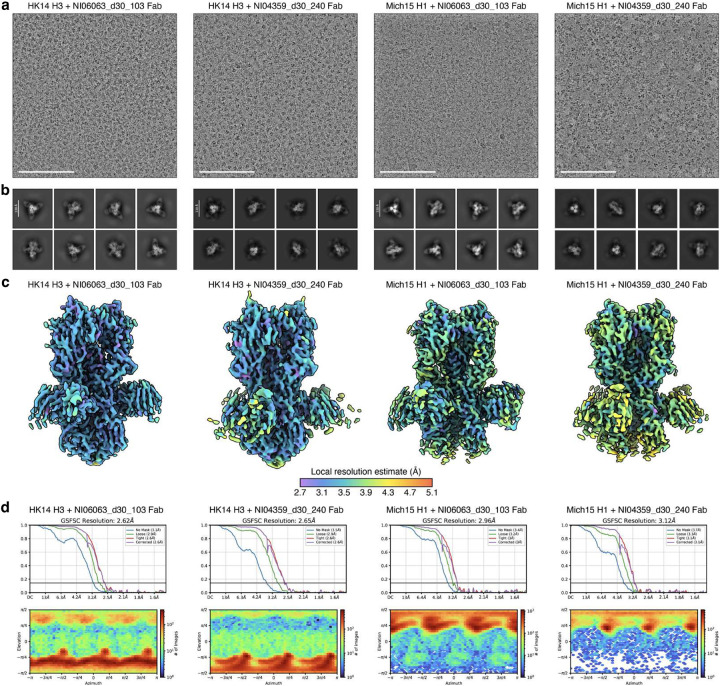
Cryo-EM data processing of NI06063_d30_103 and NI04359_d30_240 Fabs in complex with HK14 H3 and Mich15 H1. **a**,**b**, Representative micrographs (**a**) and 2D class averages (**b**) of NI06063_d30_103 and NI04359_d30_240 Fabs in complex with HK14 H3 and Mich15 H1. The scale bar represents 100 nm (**a**) or 130 Å (**b**). **c**, Local resolution maps. **d**, Gold-standard Fourier shell correlation curves and viewing direction distributions. The 0.143 cutoff is indicated by a horizontal black line.

**Extended Data Fig. 10:**
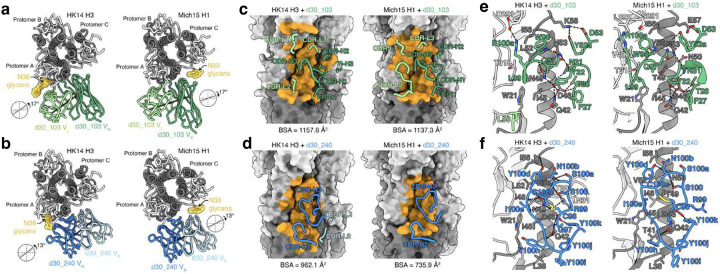
Detailed analysis of molecular interactions between HAs (HK14 H3, Mich15 H1) and H3-imprinted mAbs in Children H3-H1 group (NI06063_d30_103, NI04359_d30_240). **a**,**b**, Top-down cross-sectional views of the d30_103 (**a**) and d30_240 (**b**) complexes. The Fv domains adopt different orientations due to N38 and N33 glycans near the epitopes on HK14 H3 and Mich15 H1, respectively. Black lines connect conserved Cys residues of the heavy and light chains to visualize Fv angles and relative orientation. **c**,**d**, Epitopes of d30_103 (**c**) and d30_240 (**d**) mapped on the HA surface, with involved CDR and FR loops indicated. **e**,**f**, Detailed interactions of d30_103 (**e**) and d30_240 (**f**) around HA2 helix A. Dashed lines indicate hydrogen bonds and salt bridges.

**Fig. 5:**
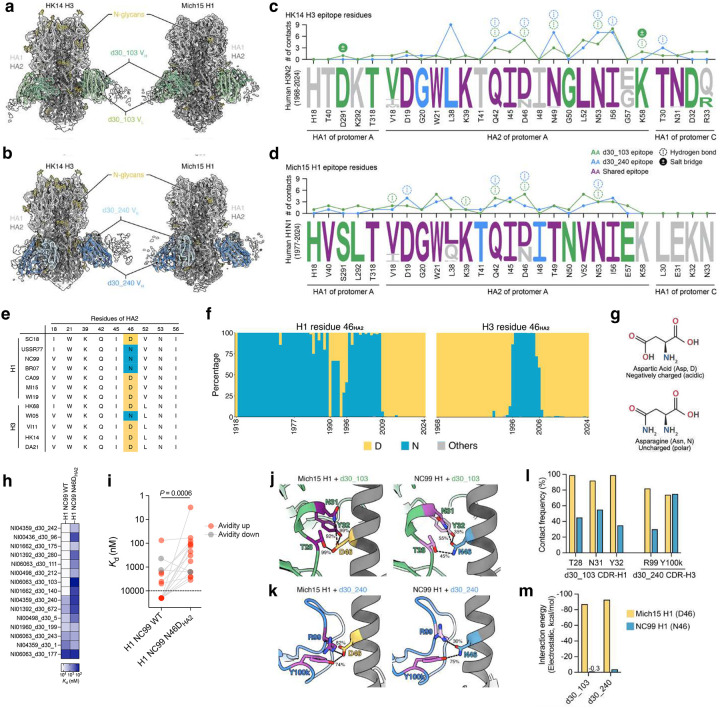
A D46N substitution in HA stalk predominantly drives heterosubtypic imprinting of cross-group reactive B cells in H3-H1 children. **a**,**b**, Cryo-EM maps and atomic models of d30_103 (**a**) and d30_240 (**b**) in complex with HK14 H3 and Mich15 H1 HAs. **c**,**d**, Epitope conservation of d30_103 and d30_240 based on human seasonal H3N2 (1968–2024; **c**) and H1N1 (1977–2024; **d**) HA sequences. Epitope residues involved in hydrogen bond or salt bridge interactions are indicated. **e**, Amino acid sequence alignment of selected HA2 residues in d30_103 epitope from multiple H1 and H3 HAs, with residue 46 highlighted in yellow (Asp, D) or blue (Asn, N). **f**, Natural occurrence frequencies of amino acid variants at position 46 of HA2 in H1 (1918–1957; 1977–2024) and H3 (1968–2024) by year. **g**, Molecular structures of residue D versus N. **h**, *K*_d_ of 15 neutralizing cross-group mAbs (IgGs) from H3-H1 children against WT NC99 H1 and NC99 H1 N46D mutant HAs. **i**, Summary data of **h** with increase or decrease in avidity indicated by color. All values higher than 10000 nM are categorized as negative and replaced by 20000 nM. Wilcoxon matched-pairs signed rank test. **j**,**k**, Interaction networks and contact probabilities between paratope residues of d30_103 (**j**) and d30_240 (**k**) and HA2 residue 46 from MD simulations of the Mich15 H1 (D46) complex and the NC99 H1 (N46) model structure. **l**,**m**, Contact frequencies (**l**) and electrostatic interaction energies (**m**) between indicated paratope residues and HA2 residue 46 from MD simulations.

To understand the molecular mechanism of H3-H1 imprinting on the HA stalk epitope, we determined the cryo-electron microscopy (cryo-EM) structures of two bnAbs, NI06063_d30_103 (d30_103) and NI04359_d30_240 (d30_240), from H3-H1 children in complex with HK14 H3 and Mich15 H1 at overall resolutions ranging from 2.62 to 3.12 Å (Extended Data Fig. 9 and Supplementary Table 3). As predicted from their binding profiles, both d30_103 and d30_240 targeted the conserved central stalk region of HA (Fig. 5a,b). Structural comparison revealed both antibodies approached HK14 H3 and Mich15 H1 at slightly different angles, accommodating the surrounding N-linked glycans in each HA with an angular shift (Extended Data Fig. 10a,b). Consistent with their higher binding affinity to HK14 H3 measured by BLI (Fig. 4b,d), both mAbs exhibited a larger buried surface area (BSA) on HK14 H3 than on Mich15 H1 and accordingly showed more extensive interactions with the major epitope region on helix A of HA2 in the HK14 H3 complex (Fig. 5c,d and Extended Data Fig. 10c–f). For both mAbs, there was an overall “better-fit” to the epitopes on H3 from the imprinting virus versus H1 from the infecting virus, partially accounting for the imprinting effects in a precursor-product manner.

We next explored whether specific epitope contact residues by cross-group mAbs from H3-H1 children might account for the shift in reactivity to pre-pdm H1N1 strains (Fig. 4b,c). Based on the conservation analysis of the d30_103 epitope, V18, L38, and D46 in HA2 were substituted in a substantial fraction of H1N1 strains circulating over the past several decades (Fig. 5d). Among the three residues, only D46 formed both a van der Waals interaction and an extensive hydrogen bond network with heavy chain residues of d30_103 that were well maintained in both H3 and H1 complexes (Fig. 5d and Supplementary Table 4). Approximately 21% of H3N2 strains (1968–2024) and 37% of H1N1 strains (1977–2024) included in the conservation analysis carry an N at this position (Fig. 5d). Single amino acid substitutions that alter antibody specificity to the cross-group stalk epitope have been observed previously^41^, including an HA2 D46N substitution that affects the binding to H3 and H1 viruses^42,43^. Notably, the pre-pdm H1N1 strains tested, including A/USSR/90/1977, A/New Caledonia/20/1999 (NC99), and A/Brisbane/59/2007, as well as an H3N2 strain (A/Wisconsin/67/2005) that mAb d30_103 failed to bind all carried an N at HA2 position 46 (Fig. 4b and Fig. 5e). Further, while most H1N1 strains before the 2009 pandemic have HA2 N46, 2009-pandemic and descendant strains all have HA2 D46 (Fig. 5f). For H3N2 strains, position HA2 46 has been predominantly fixed as a D since 2006, at least five years before the H3-H1 children were born (Fig. 5f). Together, our analyses suggest that primary infections in children by H3N2 strains with HA2 D46 in the central stalk epitope elicited MBCs that were predominantly recalled during subsequent infections with pdm H1N1 strains that also harbor HA2 D46, resulting in a shift in reactivity to pre-pdm H1N1 strains with HA2 N46. The two residues differ only in a functional group on their side chains: D has a carboxylic acid group (-COOH) and N has a carboxamide group (-CO-NH2) (Fig. 5g). While only a single group is involved, this difference likely has a disproportionate impact on antibody binding and imprinting due to the shift in charge.

**Extended Data Fig. 11:**
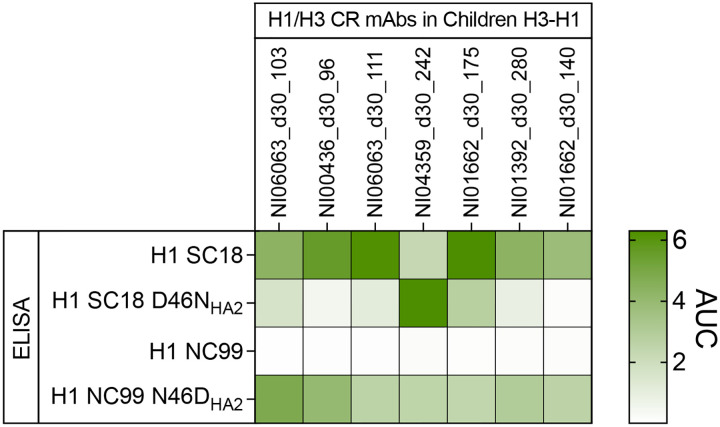
ELISA AUC of cross-group reactive mAbs from Children H3-H1 group that do not bind pre-pdm H1 HAs against WT SC18 H1, SC18 H1 D46N mutant, WT NC99 H1, and NC99 H1 N46D mutant HAs.

To validate this prediction, we introduced an HA2 D46N mutation into A/South Carolina/1/1918 (SC18) H1, which was bound by all H3-imprinted cross-group mAbs in children, and an N46D mutation into NC99 H1, which was not bound by a large fraction of those mAbs (Fig. 4b). The seven mAbs that had no binding to pre-pdm H1N1 strains were then screened against these H1 mutants by ELISA. Strikingly, six of the seven tested H3-imprinted mAbs drastically lost binding to SC18 H1 D46N, and conversely, the N46D mutation restored binding to NC99 H1 for all seven mAbs (Extended Data Fig. 11). Moreover, 87% (13/15) of the bnAbs from H3-H1 children showed increased avidity to NC99 H1 N46D compared to wild-type NC99 H1, indicating the commonality of imprinting (Fig. 5h,i). To gain mechanistic insight into the binding loss and restoration, we performed molecular dynamics (MD) simulations using our Mich15 H1 complex structures and NC99 H1 complex models generated with a previously reported NC99 H1 structure. We found that T28, N31, and Y32 in the heavy chain of d30_103 and R99 in the CDR-H3 of d30_240 showed higher contact frequencies and electrostatic interaction energies to D46 in Mich15 H1 than N46 in NC99 H1 (Fig. 5j–m). These results suggest that D46N substitution weakens the binding of H3-imprinted antibodies to the H1 stalk of pre-pdm strains by reducing favorable contacts and electrostatic interactions.

Collectively, these results demonstrate that a single atomic group difference due to a D46N substitution in the HA stalk epitope of contemporary H3N2 versus pre-pdm H1N1 virus strains predominantly drives the H3-H1 imprinting of most cross-group reactive antibodies in children, resulting in a loss of both breadth and potency in activity.

## Discussion

In this study, we investigated the origins of immune imprinting by examining B cell responses to sequential first exposures of antigenically divergent influenza virus strains in young children. Importantly, beyond being primary responses, no special phenotypic features of B cell immunity in children that predisposed them for imprinting were observed. However, these primary responses did emphasize the imprinted phenotypes of B cell responses of adults. Notably, the children’s primary responses differed in IgV gene repertoire makeup from that of adults, and there was increased cross-reactivity to past influenza virus strains in adult mAbs against both HA head and stalk epitopes. Analysis of the predicted germline antibody specificities of the cross-group B cells corroborated excessive imprinting of the adult repertoires, showing varied origins to birth year strains, including for a third to H2 that has not circulated since 1968. These distinctions between repeated exposures in adults and primary responses in children suggest that widespread homo- and heterosubtypic imprinting impact the adult B cell responses to influenza viruses.

On sequential first exposures in children, the significant antigenic distance between H1N1 and H3N2 strains precluded imprinting to most epitopes. However, the highly conserved HA central stalk epitope was preferentially targeted by cross-reactive MBCs with BCRs interacting with residues shared between H1 and H3 stalks. Affinity bias for the first infecting strains and accumulatively increased somatic mutations corroborated this finding. These imprinted B cells displayed shifts in epitope specificity, breadth, and reduced affinity and neutralization potency. For H3-imprinted B cells that were H1/H3 cross-group reactive, a single amino acid change (D46N), equating to a single atomic group side chain difference in the HA stalk, appears to have driven a nearly universal shift in specificity, and a hole in coverage of pre-pdm H1N1 strains. As this imprinting effect was observed in nearly all cross-group B cells isolated from seven children, we conclude that even slight differences in antigenicity and molecular structure can hugely impact future immune responses to particular epitopes. Thus, with a widespread focus on targeting only a few broadly conserved epitopes in the influenza vaccine field, such effects at each individual epitope are of central concern.

## Methods

### Human samples

Serum and peripheral blood mononuclear cell (PBMC) samples were obtained from participants enrolled in two prospective community-based studies in Nicaragua: The Household Influenza Transmission Study (HITS) and the Nicaraguan Pediatric Influenza Cohort Study (NPICS). HITS is an intensive transmission study that enrolls households upon identification of a laboratory-confirmed influenza case and conducts intensive follow-up of household members. NPICS is an ongoing, longitudinal cohort of children aged 0 to 14 years, established to study the development of immunity to influenza from birth through annual serosurveys and active surveillance for acute respiratory illness.

PBMCs were isolated from venous blood by density gradient centrifugation and cryopreserved for subsequent cellular immunology assays, as previously described^44^. NPICS samples were collected during 17–18, 18–19, 19–20, and 22–23 seasons, whereas HITS samples were collected during 15–16 season in Nicaragua, which typically span from June to December each year. These samples represent both pre-infection (0–3 days between symptom onset) and post-infection (4–8 weeks post symptom onset) time points, allowing for characterization of immune responses across the course of infection in a well-defined community setting. Written informed consent was obtained from parents or legal guardians of all participants, with assent from children as appropriate. Both studies received ethical approval from institutional review boards in Nicaragua and at the University of Michigan (HUM00091392 and HUM00088895).

### Cell lines and viruses

Madin-Darby canine kidney (MDCK) cells (ATCC) were maintained in culture at 37°C with 5% CO2 in Dulbecco’s modified Eagle medium (DMEM, Gibco) supplemented with 10% fetal bovine serum (FBS, Gibco), 1% l-glutamine (Gibco), and 1% penicillin-streptomycin (final concentration 100 u/mL penicillin, 100 μg/mL streptomycin, Gibco). Humanized MDCK (hCK) cells^45^ were maintained in culture at 37°C with 5% CO2 in minimum essential medium (MEM, Gibco) containing 5% (v/v) newborn calf serum (Sigma), 0.225% sodium bicarbonate (Corning), 1x amino acids (Gibco), 1x vitamins (Gibco), 1x anti-anti (Gibco), 4 mM l-glutamine (Gibco), 2 μg/mL puromycin (InvivoGen), and 10 μg/mL blasticidin (InvivoGen). Expi293F suspension cells (Thermo Fisher) were maintained in culture at 37°C with 8% CO2 in Expi293F Expression Medium (Gibco) with shaking at 125 RPM. Influenza viruses were grown in-house in MDCK cells or specific pathogen free (SPF) eggs, harvested, purified, and titered (Supplementary Table 7).

### Recombinant antigen and probe production

HA ectodomain sequences were synthesized by Integrated DNA Technologies (IDT) and cloned into a mammalian protein expression vector with a C-terminal foldon trimerization domain followed by AviTag and 6xHis tag. Constructs were transfected using ExpiFectamine 293 kit (Gibco) according to the manufacturer’s protocol. Supernatant was harvested on day 5 after transfection and incubated with Ni^2+^-nitrilotriacetic acid (Ni-NTA) agarose (Qiagen). Agarose was then loaded on a polypropylene gravity flow column (Thermo Scientific), washed with 20 mM imidazole in PBS, and eluted with a solution of 500 mM imidazole, 20 mM Tris, and 150 mM NaCl at pH 7.4. The eluate was buffer exchanged with phosphate-buffered saline (PBS) using a 100k Amicon centrifugal column (Millipore). Purified HA proteins were stored at −80°C (Supplementary Table 6).

For probes used in cell sorting, a Y98F mutation was introduced into all HA constructs, and constructs of SARS-CoV-2 WT (D614G) receptor binding domain (RBD) and chimeric HAs were generated as previously described^46–48^. All probes were synthesized, cloned, expressed, and purified as described above. The purified probes were biotinylated (AviTag-specific) with BirA enzyme following the manufacture’s protocol (Avidity). The unreacted biotin was removed by passage through a 7K molecular weight cut-off (MWCO) desalting column (Zeba spin, Thermo Fisher). Biotinylated HAs were conjugated to TotalSeq-C PE streptavidin (PE-SA, Biolegend) and BV421 streptavidin (BV421-SA, Biolegend), whereas SARS-CoV-2 WT RBD was conjugated to APC streptavidin (APC-SA, Biolegend). Chimeric HAs (cH8/1 or cH4/3) and an empty control (no antigen, PBS only) were conjugated to TotalSeq-C non-fluorescent streptavidin (NF-SA, Biolegend). The amount of antigen needed for conjugation was calculated based on a 4:1 molar ratio of antigen to a fixed amount of 0.5 μg PE-SA, BV421-SA, APC-SA, or NF-SA. Antigens were diluted in PBS to a final volume of 10 μL and SAs were added gradually to antigens 5 times on ice, 1 μL SA (0.1 mg/ml stock) every 10 minutes for a total of 5 μL (0.5 μg). The reaction was quenched with 5 μL 4mM Pierce Biotin (Thermo Fisher) for 30 minutes for a total volume of 20 μL. Probes used for each sorting experiment were prepared on the same day.

### Cell sorting for 10X Genomics single cell sequencing

PBMCs were thawed and 0.5 million cells from each subject were aliquoted. The remaining PBMCs from each subject were pooled together for B cell enrichment using EasySep^™^ pan B cell magnetic enrichment kit (STEMCELL). Enriched B cells were stained with CD19 PE-Cy7 (BD Biosciences), TotalSeq-C CD79b (Biolegend), and antigen probes (HA-PE-SA, HA-BV421-SA, RBD-APC-SA, chimeric HA/empty-NF-SA) at 1:100 dilution in FACS buffer (PBS supplemented with 2% FBS and 2 mM Pierce Biotin) on ice for 30 minutes. B cells were washed twice with FACS buffer and stained with 7-AAD Viability Staining Solution (Biolegend). Populations that were 7-AAD^−^CD19^+^APC^−^PE^+^BV421^+^ were sorted as HA-specific B cells on a BD FACSMelody cell sorter (BD Biosciences). Aliquot of 0.5 million PBMCs from each subject was stained with CD19 PE-Cy7, CD4 BB515 (BD Biosciences), and a unique TotalSeq-C anti-human hashtag (Biolegend) at 1:100 dilution in FACS buffer on ice for 30 minutes. Aliquots were washed twice with FACS buffer and subsequently pooled together before staining with 7-AAD. Populations that were 7-AAD^−^CD19^+^ were sorted as CD19+ B cells. To account for low cell numbers, several thousands of CD4+ T cells from pooled PBMCs were sorted and mixed into HA-specific B cells as carrier cells. PBMCs from acute or convalescent time points were processed and sorted separately.

Sorted cells were immediately loaded on a 10X Chromium X Controller to generate single-cell gel-beads in emulsion (GEM) using Chromium Next GEM Single Cell 5’ HT Reagent kits v2 (10X Genomics). 10X single cell libraries of 5’ Gene Expression, V(D)J BCR, and Feature Barcode (cell surface protein) were prepared following manufacturer’s user guide (CG000424 Rev B). Libraries were pooled and sequenced using NextSeq 1000/2000 P2 XLEAP-SBS Reagent Kit (300 cycles) on a NextSeq 1000 platform (Illumina) with the configuration of 26 cycles for read 1, 10 cycles for i7/i5 index, and 150 cycles for read 2.

### Computational analysis for single cell sequencing data

Libraries of 5’ Gene Expression, V(D)J BCR, and Feature Barcode were demultiplexed using the cellranger mkfastq pipeline. FASTQ reads were mapped to the human genome (GRCh38–2020-A) either using a combination of cellranger_count and cellranger_vdj, or cellranger_multi (v7.0.1 or newer). Downstream analyses were performed in R v4.2.2 using Seurat (v4.3.0 or newer), including quality control, data normalization, data scaling, dimension reduction (both linear and non-linear), clustering, differential expression analysis, batch effects correction, data visualization, and uniform manifold approximation and projection (UMAP) generation. Quality control thresholds for each dataset were determined based on the distributions of detectable gene numbers (remove outliers with < 200 or > 3000–5000 based on dataset) and percentage of mitochondrial genes (remove outliers with > 5–10% based on dataset). Donor identity of each cell was determined using an in-house hybrid demultiplexing approach that integrated results of cell hashing (hashtag) and a single nucleotide polymorphisms-based demultiplexing method (Souporcell) as previously described^49^. The following cells were filtered out: cells classified as doublets or unassigned based on hybrid demultiplexing; outliers in the distribution of detectable gene numbers or percentage of mitochondrial genes; and cells not expressing CD79A or expressing any of the non-B cell markers (LYZ, CD14, CD8A, GNLY, PPBP, CD3E, FCER1A, FCGR3A). Transcriptome data were normalized by a log-transform function with a scaling factor of 10,000 whereas cell surface protein data (TotalSeq antibody and antigen probe) were normalized by a centered log-ratio (CLR) normalization. Batch effects correction analysis was performed using an Anchor method implemented in Seurat. Following integration, we performed data scaling and linear dimension reduction using variable genes in principal component analysis (PCA) and the top 15 principal components (PCs). High-quality cells were then clustered by Louvain algorithm implemented in Seurat under the resolution of 0.6. Cells were clustered using FindNeighbors and FindClusters functions. Further dimension reduction and visualization were performed with RunUMAP function. Differentially expressed genes for each cell cluster were identified using a Wilcoxon Rank Sum test implemented in Seurat. B cell subset identity was assigned to each cluster based on differentially expressed genes (DEGs) and expression of stereotypical gene markers. We performed Gene Set Enrichment Analysis (GSEA) with DEGs from a putative recent GC emigrant cluster using the fgsea package v1.24.0. Gene sets from a published study^36^ were used for GSEA and relevant biological processes with adjusted P value < 0.01 and normalized enrichment score > 2 were plotted (Extended Data Fig. 3e).

### Single cell BCR sequencing analysis

Full-length V(D)J contigs were assembled with cellranger_vdj or cellranger_multi (v7.0.1 or newer) and aligned to IMGT reference using IgBLAST v1.20.0. The downstream analyses and annotations were done using an in-house single-cell multi-model analysis software VGenes (https://wilsonimmunologylab.github.io/VGenes/). Cells with ‘good BCR’ were filtered using the following criteria: cells passed the transcriptome quality control, V(D)J information is available for one heavy chain paired with one light chain, classified as full-length and productive by VGenes. Chord plots for B cell repertoires were generated in R using chordDiagram function of circlize package v0.4.16.

### Antigen specificity prediction via probe score

Unnormalized unique molecular identifier (UMI) counts were extracted from the Seurat ADT assay for cells with ‘good BCR’. A probe score for each antigen was calculated as the UMI ratio of antigen to CD79b and an empirical cutoff of 0.1 was set to distinguish positive from negative. UMI ratios shown in probe score heatmaps were log2 transformed for better visualization. To account for the dilution effect in UMI per antigen for cells reactive to multiple antigens, cells were considered positive for a given subtype (H1 or H3) when the sums of probe scores of all HAs from the same subtype were higher than 0.1. Subtype reactivity of each cell was determined based on the following criteria: (1) Cells positive for H1 were identified as H1 reactive; (2) Cells positive for H3 were identified as H3 reactive; (3) Cells with UMI > 0 for at least 2 antigens from each subtype and positive for both H1 and H3 were identified as H1/H3 cross-reactive; (4) Cells positive for the empty probe (no antigen) were identified as polyreactive; (5) Cells positive for neither H1 or H3 were identified as nonreactive. Cells belonging to the polyreactive, or nonreactive category, were considered non-specific and removed from the dataset of HA-specific B cells. Within H1 reactive cells, stalk binders were identified when scores of chimeric H1 (cH8/1) were higher than the mean scores plus 3 standard deviations of all H1s, while head binders were identified otherwise. The predicted antigen specificities of subsets of B cells were validated with corresponding mAbs by ELISA.

### Serum ELISA

High protein-binding microtiter plates (Costar) were coated with recombinant HA antigens at 2 μg/mL in PBS overnight at 4°C. Plates were washed 3 times with PBS containing 0.05% Tween 20 (PBS-T) and incubated with 200 μL blocking buffer (PBS containing 0.1% Tween 20 and 3% skim milk powder) for 1 hour at room temperature (RT). Serums were 3-fold serially diluted in dilution buffer (PBS containing 0.1% Tween 20 and 1% skim milk powder) starting at 1:100. Plates were incubated with 100 μL serum dilutions for 2 hours at RT. Horseradish peroxidase (HRP)-conjugated goat anti-human IgG secondary antibody (Sigma-Aldrich) was diluted at 1:3000 in dilution buffer. Plates were incubated with 100 μL secondary antibody dilutions for 1 hour at RT. Plates were washed 3 times with PBS-T and developed with 100 μL SigmaFast o-phenylenediamine dihydrochloride (OPD) solution (Sigma-Aldrich) for 10 minutes in the dark. Additional 50 μL 3M hydrochloric acid was added to stop the development reaction. Absorbance was measured at 490 nm on a microplate spectrophotometer (Bio-Rad). Area under the curve (AUC) for each serum sample was calculated in GraphPad Prism 10. Limit of detection (LOD) was calculated as mean absorbance plus 3 standard deviations of serums at acute time point from H1 only children who were previously naïve to influenza. All AUCs below LOD were replaced by a fixed value of 15.

### Recombinant monoclonal antibody and Fab production

Subsets of HA-specific B cell clones with ‘good BCR’ from different cohort groups or time points were randomly selected for monoclonal antibody (mAb) expression. Notably, all H1/H3 cross-reactive clones were selected to enrich sample size for memory recall response. The immunoglobulin heavy and light chain sequences of these clones were synthesized as gene fragments by Integrated DNA Technologies (IDT) or Twist Bioscience. To generate germline version of mAbs from adults, the somatic mutations within V regions were reverted for both heavy and light chain sequences. Synthesized gene fragments were cloned into human IgG1, human kappa, or human lambda expression vectors. The heavy and light chain constructs of each mAb were transiently co-transfected into Expi293F cells using polyethylenimine (PEI, Polyscience). The supernatants containing secreted mAbs were harvested on day 5 and purified using protein A-agarose beads (Thermo Fisher) as described previously^50^. To generate Fab, the heavy chain fragments of selected mAbs were inserted into a modified human IgG1 vector with Fc constant region replaced by 6xHis tag. The Fab version heavy chain and corresponding light chain constructs were co-transfected and secreted Fabs within supernatants were purified following the same protocol of purifying recombinant antigen as described above, except that the buffer exchange was done using a 10k Amicon centrifugal column.

### Antigen-specific high avidity ELISA

High-protein binding microtiter plates were coated with recombinant mAbs at 2 μg/ml in PBS overnight at 4°C. Plates were washed 3 times with PBS-T and blocked with 150 μL PBS containing 20% FBS for 1 hour at 37°C. Antigen probes were generated as described before with unconjugated streptavidin (Invitrogen) and serially diluted 1:3 starting at 30 μg/ml. Plates were incubated with 50 μL of probe dilutions for 1 hour at 37°C. Horseradish peroxidase (HRP)-conjugated rabbit anti-streptavidin polyclonal antibody was diluted 1:1000 (Abcam) and 70 μL of antibody dilution was used to detect binding of probes. Plates were washed 3 times with PBS-T and subsequently developed with Super Aquablue substrate (eBiosciences). Absorbance was measured at 405 nm on a microplate spectrophotometer (BioRad). Control antibodies with known binding characteristics were included on each plate and results were recorded when the absorbance of control reached 1.0 optical density (OD) units.

### Focus reduction neutralization test

The day before the experiment, 25,000 MDCK cells were added to each well of a 96-well plate. Serial 3-fold dilutions of mAb in 50 μl MEM, starting at 200 μg/ml, were mixed with an equal volume of 100 focus-forming units of virus for 1 hour at 37 °C and added to MDCK cells. Overlay was prepared by mixing 50 μl 2% methylcellulose (Sigma) and an equal volume of mAb dilutions in MEM supplemented with 1 μg/ml tosyl phenylalanyl chloromethyl ketone (TPCK)-treated trypsin. The virus-antibody mixture was removed after 1 hour of incubation. The 100 μl overlay was added to cells and plates were incubated for 20 hours at 37 °C. Cells were washed with PBS, fixed with 100 μl 80% ice-cold acetone at −20 °C for at least 1 hour, and blocked for 30 min with 100 μl staining buffer (PBS containing 3% FBS). Cells were incubated with 100 μl mouse anti-NP antibody (clone A3, Millipore), followed by 100 μl goat anti-mouse IgG HRP (Southern Biotech) diluted 1:1000 in staining buffer for 1 hour at room temperature. The plates were washed 3 times with PBS and developed with 50 μl TrueBlue Peroxidase Substrate (SeraCare Life Sciences) in the dark for 5 minutes. Quantification of viral foci was performed using an S6 Universal M2 reader (ImmunoSpot). The inhibition percentage was calculated as: ((Virus only foci – Sample foci)/Virus only foci) × 100. The half-maximal inhibitory concentration (IC50) or half-maximal focus reduction neutralization (FRNT50) for mAbs was determined using the log(inhibitor) versus normalized response (variable slope) analysis in GraphPad Prism 10.

### Biolayer interferometry

MI15 H1, HK14 H3, NC99 H1, or NC99 H1 N46D were biotinylated and loaded at a concentration of 40 μg/ml onto streptavidin biosensor (Forte Bio/Sartorius) for 300 seconds. After the sensor was equilibrated in kinetic buffer (PBS containing 0.02% Tween 20 and 0.1% BSA) for 60 seconds, the sensor was first soaked into 500 nM Fab or IgG for 150 seconds as association step and then soaked into kinetic buffer for 150 seconds as dissociation step. The equilibrium dissociation constant (Kd) of each Fab or IgG was calculated using Octet Analysis Software 12.2.2.26 (Forte Bio/Sartorius). Results with coefficient of determination (R^2^) < 0.96 were removed and considered negative (no binding).

### Generation of viral mutants

The HA sequences of five H1N1 A/Michigan/45/2015 mutants each with one major antigenic site (Sa, Sb, Ca1, Ca2, Cb) ablated were obtained from a previous study^51^. Influenza eight-plasmid reverse genetics system^52^ was used to construct the mutant viruses. Briefly, DNA plasmids of hemagglutinin (HA) mutants were cloned into a pHW2000 vector and transfected into cocultured MDCK-SIAT cells and human embryonic kidney 293T cells with the other seven influenza segments of PR8. After 72 hours, supernatants were collected. Viruses were plaque purified on MDCK-SIAT cells grown in DMEM (Gibco) containing 10% FBS (Gibco) and penicillin-streptomycin mix (Gibco). Individual plaques were picked and grown in fresh MDCK-SIAT cell. To confirm the HA sequences of the viral mutants, viral RNAs were extracted from the supernatant and HA segments were amplified and confirmed by Sanger sequencing.

### Hemagglutination inhibition

A/Michigan/45/2015 H1N1 wild-type or mutant viruses were diluted to eight HA units per 50 μl. Then 25 μl of the diluted virus was combined in duplicate wells with an equal volume of serially diluted monoclonal antibodies, starting from 100 μg/mL in PBS. The virus-antibody mixture was incubated for 30 min at room temperature. 25 μl of 1% turkey red blood cells (Lampire) was then added and incubated for 30 min at room temperature. Minimum effective concentrations were read based on the final dilution at which hemagglutination was observed. For the assay using A/Victoria/361/2011 H3N2 virus, the same protocol was followed using guinea pig red blood cells (Lampire) with monoclonal antibodies starting from 60 μg/mL.

### Deep mutational scanning

The HA1 mutant library was generated in the background of H1N1 A/Michigan/45/2015 (Genbank accession number MK622940.1, residues L87-G254) via saturation mutagenesis. The linearized vector was generated from a pCTcon2 yeast display plasmid by using 5′-GGC CGG CTG GGC CGC TGC TAA AAC TGA AGC AAT AAC AGA A-3′ and 5′-GGC CTC GGG GGC CTG TAC CCA TAC GAC GTT CCA GAC TAC G-3′ as primers. Inserts were generated by two batches of PCRs, followed by overlapping PCRs. The first batch of PCRs consisted of 21 reactions, each with an equal molar mix of eight primers as the forward primer and a universal reverse primer 5′-GCC TTC GCC GGA GCC TGG CTT GC-3′. The forward primers for the first batch of PCRs are listed in Supplementary Table 8. These forward primers were named as cassetteX_N, in which X represents the cassette ID and N represents the primer number. Forward primers with the same cassette ID were mixed at equal molar ratio and used in the same PCR. The second batch of PCRs consisted of another 21 reactions, each with a universal forward primer 5′-ACC TCT ATA CTT TAA CGT CAA GG-3′ and a unique reverse primer as listed in Supplementary Table 8. Subsequently, 21 overlapping PCRs were performed using the universal forward primer and the universal reverse primer. For each overlapping PCR, the template was a mixture of 10 ng each of the corresponding products from the first and second batches of PCRs. The complete insert was an equal molar mix of the products of these 21 overlapping PCRs. All PCRs were performed using PrimeSTAR Max polymerase (Takara Bio) according to the manufacturer’s instructions. PCR products were purified using Monarch DNA Gel Extraction Kit (New England Biolabs).

Yeast cells were transformed by electroporation following a previously described protocol^53^. Briefly, 5 μg of the HA1 insert library and 4 μg of the corresponding purified linearized vector were added into 400 μL of conditioned yeast. The mixture was electroporated and transferred into Yeast Peptone Dextrose (YPD) media supplemented with 4 mL of 1 M sorbitol and incubated at 30 °C with shaking at 225 rpm for 1 h. Cells were plated onto Synthetic Dextrose Casamino Acid (SD-CAA) plates and incubated at 30 °C for 40 h. Colonies were then collected in SD-CAA medium, centrifuged at 1700 rcf for 5 min at room temperature, and resuspended in SD-CAA medium with 15% v/v glycerol such that OD600 was 50. Glycerol stocks were stored at −80 °C until used.

Glycerol stock of the yeast display library was recovered in SD-CAA medium and induced in Synthetic Galactose Raffinose Casamino Acid (SGR-CAA) medium. We initially used APC anti-HA.11 (epitope 16B12, BioLegend) at a final concentration of 1 μg/mL to sort the yeast cells on expression level. Following the positive gating on the expression sort, PE-conjugated monoclonal antibodies were used at a final concentration of 10 μg/mL to gate the yeast cells for binding sort of HA1 variant library. Using a BD FACSMelody^™^ Cell Sorter (BD Biosciences), yeast cells were gated by no HA1 expression (PE^−^APC^−^), HA1 expression with no binding (APC^+^PE^−^), HA1 expression with binding (PE^+^APC^+^). The sorted cells were recovered in SD-CAA medium. Frozen stocks were made and stored at −80°C until used. FlowJo v10.8 software (BD Life Sciences) was used to analyze FACS data.

Plasmids from the yeast cells were extracted using a Zymoprep Yeast Plasmid Miniprep II Kit (Zymo Research) following the manufacturer’s protocol. The HA1 mutant library was amplified by PCR using forward recovered primers 5’- CAC TCT TTC CCT ACA CGA CGC TCT TCC GAT CTT CCT GGG AAA TCC AGA GTG TGA A-3’ and reverse recovered primers 5’- GAC TGG AGT TCA GAC GTG TGC TCT TCC GAT CTC CAG TTG CTT CGA ATG TTA TTT T-3’. Subsequently, adapters containing sequencing barcodes were appended to the amplicon using primers 5’-AAT GAT ACG GCG ACC ACC GAG ATC TAC ACX XXX XXX XAC ACT CTT TCC CTA CAC GAC GCT-3’, and 5’-CAA GCA GAA GAC GGC ATA CGA GAT XXX XXX XXG TGA CTG GAG TTC AGA CGT GTG CT-3’. Positions annotated by an “X” represented the nucleotides for the index sequence. All PCRs were performed using Q5 High-Fidelity DNA polymerase (NEB) according to the manufacturer’s instructions. PCR products were purified using PureLink PCR Purification Kit (Thermo Fisher Scientific). The final PCR products were submitted for next generation sequencing using MiSeq v3 PE300 (Illumina).

Next-generation sequencing data were obtained in FASTQ format. Forward and reverse reads of each pair-end read were merged by PEAR^54^. The merged reads were parsed by SeqIO module in BioPython^55^. Primer sequences were trimmed from the merged reads. Trimmed reads with lengths inconsistent with the expected length were discarded. The trimmed reads were then translated to amino acid sequences, with sequencing error correction performed at the same time as previously described^56^. The number of reads corresponding to each HA1 variant in each sample is counted. Frequency (F) of a mutant *i* at position *s* within bin *n* of replicate *k* was computed for each replicate as follows:

$$F_{i,s,n,k}=\frac{{\text{readcount}}_{i,s,n,k}+1}{\sum_{s}\sum_{i}\left({\text{readcount}}_{i,s,n,k}+1\right)}$$

A pseudocount of 1 was added to the read counts of each mutant to avoid division by zero in subsequent steps. We then calculated the HA1 expression ratio (ER) of a mutant *i* at position *s* of replicate *k* as follows:

$$ER_{i,s,k}=\frac{F_{PE+(i,s,k)}}{F_{PE+(i,s,k)}+F_{PE-(i,s,k)}}$$

Subsequently, we calculated the escape score (ES) of each mutant *i* at position *s* of replicate *k* as follow:

$$ES_{i,s,k}=\frac{F_{APC+(i,s,k)}}{F_{PE+(i,s,k)}}\times ER_{i,s,k}$$

The expression score of a mutant *i* at position *s* of was then calculated by taking the average of the expression scores between replicates.

In the HA1 yeast display library, structural destabilizing mutations could potentially impact the interaction between HA and all three tested antibodies. This would lead to high escape score for some mutations, despite not being part of the epitope^57^. Two antibodies Hb-9 and Hb-5 have been used as control in the experiments. Of note, antibody Hb-5 binds to Sa only and Hb-9 doesn’t bind to any of five immunodominant sites. Based on our mutant virus HAI experiments, we inferred that their binding epitopes differed from one another. Therefore, to mitigate the effects of these destabilizing mutations, we determined the final escape score (FES) of each mutant *i* at position *s* of replicate *k* by subtracting its average escape score against all five tested antibodies, as destabilizing mutations are more likely to exhibit high escape score across all five screens:

$$FES_{i,s}=ES_{i,s}-\frac{ES_{\text{mAb-}129\;(i,s)}+ES_{\text{mAb-}235\;(i,s)}+ES_{\text{mAb-}687\;(i,s)}+ES_{\text{mAb-Hb-}5\;(i,s)}+ES_{\text{mAb-Hb-}9\;(i,s)}}{5}$$

To overlay the epitope of each antibody onto the HA structure, the average final escape score of position *s* was then calculated by taking the average of the final escape score of all mutants at that position:

$$FES_{s}=\frac{\sum_{i\in s}FES_{i,s}}{\left|\left\{i\in s\right\}\right|}$$

The average final escape scores of each antibody were then projected onto the cryo-EM structure of A/Michigan/45/2015 HA (PDB: 6XGC).

### Microneutralization

Two-fold serial dilutions of the mAbs (ranging from 480 μg/mL to 0.23 μg/mL) were performed in MEM supplemented with 0.3% (v/v) BSA and 0.00006% (v/v) TPCK. Each dilution was mixed with equal volume of 100 tissue culture infectious doses 50 (TCID50) of virus (so that the final mAbs concentrations range from 240 μg/mL to 0.12 μg/mL) and incubated at 37°C for 1 hour. The mAb-virus mixtures were inoculated into MDCK cells (for avian viruses and H1N1 viruses) or hCK cells (for H3N2 viruses) in quadruplicates in a 96-well plate and incubated at 39°C (for avian viruses) or 37°C (For H1N1 and H3N2 viruses) with 5% CO2 for 5 days. Inhibitory concentration 50 (IC50) values were calculated according to the Reed and Muench method based on the observed cytopathic effect in the inoculated cells.

### Cryo-EM sample preparation and data collection

Purified d30_103 and d30_240 Fabs were mixed with HK14 H3 HA and Mich15 H1 HA, respectively, at a molar ratio of 1:2 (HA protomer:Fab), and incubated at room temperature for 1 hour before grid preparation. The final concentration of HA in each sample ranged from 0.7 to 0.8 mg/mL. Samples were mixed with octyl-β-glucoside (OBG; final concentration 0.1% w/v) to aid particle tumbling and applied to Quantifoil 1.2/1.3 300 mesh copper grids. Grids were plunge-frozen in liquid ethane using a Vitrobot Mark IV (Thermo Fisher Scientific) with a blot force of 1 and a blot time of 4–4.5 seconds at 100% humidity and 4°C. Micrographs for the d30_103-HK14 H3 and d30_240-HK14 H3 complexes were collected on a Glacios 2 microscope (Thermo Fisher Scientific) operating at 200 kV with a Falcon 4i direct electron detector, while micrographs for the d30_103-Mich15 H1 and d30_240-Mich15 H1 complexes were collected on a Glacios microscope equipped with a Falcon IV detector. Automated data collection was carried out using EPU (Thermo Fisher Scientific) at a nominal magnification of 190,000, with a total exposure dose of ~45 e-/Å2. The pixel size was 0.718 Å for the HK14 H3 datasets and 0.725 Å for the Mich15 H1 datasets, with a nominal defocus range of −0.6 to −1.4 μm and −0.7 to −1.4 μm, respectively.

The purified H1N1 A/Solomon Islands/3/2006 HA protein was mixed with NI04359_d30_245 and NI01056_d30_604 Fab at 1:4 molar ratio (Supplementary Table 9) and incubated overnight at 4°C before purifying by size exclusion chromatography on the Superose 6 Increased 10/300 column (Cytiva) in 20 mM Tris-HCl pH 8.0 and 100 mM NaCl. The anchor Fab FISW84^58^ was also added into the complex of NI01056_d30_604 Fab and H1N1 A/Solomon Islands/3/2006 HA to reduce the preferred orientation of the complex. The complex peak was concentrated to ~3 mg/mL and mixed with 0.5% w/v n-octyl-ß-D-glucoside (Anagrade) to a final concentration of 0.1% w/v just before loading on the grid. Cryo-EM grids were prepared using a Vitrobot Mark IV machine. An aliquot of 3 μL sample was applied to a 300-mesh Quantifoil R1.2/1.3 Cu grid pretreated with glow-discharge. High-resolution cryo-EM movies were collected on an FEI Titan Krios at 300 kV with a Gatan K3 detector.

### Cryo-EM data processing and model building

All datasets of d30_103 and d30_240 were processed using cryoSPARC^59^. Dose-weighted movie frame alignment was carried out using Patch motion correction in cryoSPARC live to account for stage drift and beam-induced motion. The contrast transfer function (CTF) was estimated using Patch CTF in cryoSPARC live. Micrographs were curated based on CTF fits, with those worse than 7–8 Å excluded due to poor quality. Individual particles were selected from an initial subset of several hundred micrographs using Blob picker. After several rounds of 2D classification, classes resembling the Fab-HA complex were used to pick templates for all micrographs. Clean particle stacks were selected through iterative rounds of 2D classification and subsequently used for ab initio reconstruction, followed by heterogeneous refinement to further remove junk particles. The resulting reference volume was used as an initial model for homogeneous and/or non-uniform refinement with C3 symmetry applied. Particles were subjected to 3D classification, and the best classes were further refined using non-uniform refinement with subsequent global CTF refinement, yielding the final map. For model building, the AlphaFold3-predicted model and the cryo-EM structure of Mich15 H1 (PDB: 7KNA) were used as the initial models for HK14 H3 and Mich15 H1, respectively. The antibody Fv models were generated using ABodyBuilder2^60^. The models were fitted into the cryo-EM maps using UCSF ChimeraX^61^. The models were manually adjusted using Coot^62^ and further refined through Rosetta Relax^63^ and real-space refinement in Phenix^64^. HAs were numbered according to the H3 numbering scheme, while the antibody Fv was numbered based on the Kabat numbering scheme. Buried surface area (BSA), epitope and paratope residues, and their interactions were analyzed using the PISA server^65^ and Epitope Analyzer^66^. BSA was calculated using a cutoff of >5 Å2, and hydrogen bonds were defined as atomic interactions with a distance of ≤3.5 Å. Structural figures were generated using UCSF ChimeraX^61^.

Datasets of NI04359_d30_245 and NI01056_d30_604 were processed with CryoSPARC^59^ (version 4.5). Movies were subjected to motion correction and CTF estimation, and particles were picked with CryoSPARC blob picker followed by 2D classification^67^. Best classes from blob picker were used as templates for CryoSPARC template pickers, and the resulting particles were cleaned up by multiple rounds of 2D classification before ab initio reconstruction. The best class from ab initio reconstruction was subjected to homogenous refinement, reference-based motion correction, another round of homogenous refinement, local and global CTF estimation, and non-uniform refinement. Antibody NI04359_d30_245 and NI01056_d30_604 complexes were processed with C1 symmetry. All maps were sharpened with DeepEMhancer^68^ and all initial atomic model were built using ModelAngelo^69^. The models were subjected to multiple rounds of manual refinement in Coot^70^ (version 0.9.8) and real-space refinement in Phenix^64^. This process was iterated for several cycles until no significant improvement of the model was observed. Validation statistics are listed in Supplementary Table 9.

### HA sequence conservation analysis

Full-length human H3N2 and H1N1 HA protein sequences circulating from 1968 to 2024 and 1977 to 2024, respectively, were downloaded from GISAID database. To minimize temporal sampling bias, up to 10 sequences per year were selected. Multiple sequence alignments were conducted using Clustal Omega, and the resulting alignments were used to generate sequence logos with WebLogo 3. The sequence logos were further manually refined and annotated in Adobe Illustrator.

All human H1 (1918–1957; 1977–2024) and H3 (1968–2024) protein sequences with complete HA2 regions were downloaded from NCBI and GISAID (as of September 22, 2024). Using a Python script, the natural occurrence frequencies of amino acid at position 46 of HA2 in each year were calculated for H1 and H3 separately. The results were plotted as bar graphs with the ggplot2 package in R (version 4.2).

### Molecular dynamics simulation

Cryo-EM structures of d30_103 and d30_240 in complex with Mich15 H1 were used as starting models for molecular dynamics (MD) simulations. In addition, complexes of d30_103 and d30_240 with NC99 H1 were modeled based on the previously reported NC99 H1 structure (PDB 8TP5) and the Mich15 H1 complexes. Starting structures for MD simulations were prepared in the Molecular Operating Environment (Chemical Computing Group) using the Protonate3D tool^71^. These structures were then further processed using CHARMM-GUI^72^. Each model was placed in a cubic water box of TIP3P water molecules with a minimum wall distance of 12 Å from the protein, and the system was neutralized with K^+^ and Cl^−^ ions to a final concentration of 0.15 mM^73^. For all simulations, parameters of the AMBER force field 19SB were used^74^. For the glycans, we used the GLYCAM force field, namely the GLYCAM-06j parameter set^75^. We then performed 3 repetitions of 1 μs of classical molecular dynamics simulations for each HA-antibody complex using Amber24^76^. MD simulations were performed in an NpT ensemble using pmemd.cuda^77^. Bonds involving hydrogen atoms were restrained by applying the SHAKE algorithm, allowing a time step of 2 fs^78^. The Langevin thermostat was used to maintain the temperature during simulations at 300 K with a collision frequency of 2 ps^−1^, and a Monte Carlo barostat with one volume change attempt per 100 steps was applied^79–81^. The interaction energies were calculated with cpptraj using the interaction energy (LIE) tool^82^. The electrostatic interaction energies were calculated for all frames of each simulation and provided the simulation-averages of these interactions. To calculate the interactions and interaction frequencies of the binding interface, we used the GetContacts tool (Stanford University, https://getcontacts.github.io/). ChimeraX was used for visualization^61^.

### Statistical analysis

All statistical analyses were performed using R v4.2.2 or GraphPad Prism 10. Details of the statistics used in each experiment were indicated in the corresponding figure legends. P values lower than 0.05 were considered significant (* P < 0.05; ** P < 0.01; *** P < 0.001; **** P < 0.0001), while P values higher than 0.05 were considered non-significant (n.s.). The number of biological repeats for experiments and specific tests for statistical significance used are indicated in the figure legends.

## Extended Data

## Supplementary Material

1 Supplementary Table 1: Clinical data and exposure history of adults and children.
Supplementary Table 2: Antigen probes for sorting HA-specific B cells.
Supplementary Table 3: Cryo-EM data collection, refinement and validation statistics for NI06063_d30_103 and NI04359_d30_240.
Supplementary Table 4: Intermolecular contacts of NI06063_d30_103 with HK14 H3 and Mich15 H1.
Supplementary Table 5: Intermolecular contacts of NI04359_d30_240 with HK14 H3 and Mich15 H1.
Supplementary Table 6: Recombinant HA proteins and assays they are involved with.
Supplementary Table 7: Influenza virus strains and assays they are involved with.
Supplementary Table 8: PCR primers used in deep mutational scanning.
Supplementary Table 9: Cryo-EM data collection, refinement and validation statistics for NI04359_d30_245 and NI01056_d30_604.

Supplementary Information is available for this paper.

This is a list of supplementary files associated with this preprint. Click to download.

- PDBvalidationreportscombined.pdf

## Data Availability

The cryo-EM maps and atomic coordinates have been deposited to the Electron Microscopy Data Bank (EMDB) and the PDB with accession numbers: EMD-70233 and 9O8Q for NI06063_d30_103 Fab or EMD-70235 and 9O8S for NI04359_d30_240 Fab in complex with HA of A/Hongkong/4801/2014; EMD-70234 and 9O8R for NI06063_d30_103 Fab or EMD-70236 and 9O8T for NI04359_d30_240 Fab in complex with HA of A/Michigan/45/2015; EMD-70502 and 9OI2 for NI04359_d30_245 Fab or EMD-70503 and 9OI3 for NI01056_d30_604 Fab in complex with HA of A/Solomon Islands/3/2006. All 10X scRNA-seq data generated in this study (5’ transcriptome, B cell receptor immunoglobulin VDJ gene repertoire, and cell surface feature barcode) are available in the Gene Expression Omnibus database (GEO) under the GEO Series record GSE297740. All datasets generated and information presented in the study are available from the corresponding authors on reasonable request. Materials generated in this study can be made available on request and may require a material transfer agreement.
