# Supplementary material for: A single residue change only differing by an atomic group can drive imprinting to influenza
Source: Res Sq. 2025 Jul 7:rs.3.rs-6914018. Preprint. [Version 1] doi: 10.21203/rs.3.rs-6914018/v1 (PMC12265162; doi:10.21203/rs.3.rs-6914018/v1)
Supplement: 1 — Supplementary Table 1: Clinical data and exposure history of adults and children. Supplementary Table 2: Antigen probes for sorting HA-specific B cells. Supplementary Table 3: Cryo-EM data collection, refinement and validation statistics for NI06063_d30_103 and NI04359_d30_240. Supplementary Table 4: Intermolecular contacts of NI06063_d30_103 with HK14 H3 and Mich15 H1. Supplementary Table 5: Intermolecular contacts of NI04359_d30_240 with HK14 H3 and Mich15 H1. Supplementary Table 6: Recombinant HA proteins and assays they are involved with. Supplementary Table 7: Influenza virus strains and assays they are involved with. Supplementary Table 8: PCR primers used in deep mutational scanning. Supplementary Table 9: Cryo-EM data collection, refinement and validation statistics for NI04359_d30_245 and NI01056_d30_604. [file NIHPPRS6914018V1-supplement-1.pdf]

Supplementary Table 1: Clinical data and exposure history of adults and children.

Subject	Group	Age	Sex	H3 season	H1 season	Acute d.p.i.	Conv. d.p.i.	Acute PBMCs	Conv. PBMCs
NI02281	Adults H1	36	F	N/A	2015-Nov (PCR)	0	32	3.9M	3.9M
NI01403	Adults H1	43	M	N/A	2015-Nov (PCR)	0	31	3.6M	2.4M
NI00476	Adults H1	21	F	N/A	2015-Nov (PCR)	0	57	2.1M	6.9M
NI01056	Adults H1	19	M	N/A	2015-Nov (PCR)	2	33	1.5M	6.3M
NI06165	Adults H1	27	F	N/A	2015-Nov (PCR)	-2	44	4.8M	4.5M
NI05755	Adults H1	26	F	N/A	2015-Nov (PCR)	-1	44	2.1M	3.3M
NI04525	Adults H1	46	M	N/A	2015-Nov (PCR)	-2	41	3.3M	2.7M
NI03831	Adults H1	36	M	N/A	2015-Nov (PCR)	0	38	1.2M	3.9M
NI01387	Adults H1	43	F	N/A	2015-Nov (PCR)	0	30	1.2M	3.6M
NI06057	Adults H1	23	F	N/A	2015-Nov (PCR)	0	30	1.8M	6.6M
NI00337	Adults H1	49	F	N/A	2015-Nov (PCR)	0	30	N/A	3M
NI02255	Adults H1	32	F	N/A	2015-Nov (PCR)	0	33	3.3M	6.3M
NI04004	Children H1 only	0	M	N/A	2019-Oct (PCR)	2	35	7.5M	6.5M
NI04189	Children H1 only	0	M	N/A	2023-Jun (PCR)	5	44	5M	3M
NI01686	Children H1 only	3	M	N/A	2015-Nov (PCR)	-2	28	2.5M	6M
NI01662	Children H3-H1	4	F	2011-Nov (PCR)	2015-Nov (PCR)	2	32	N/A	9.9M
NI01960	Children H3-H1	2	F	Serology	2015-Nov (PCR)	0	43	3M	11.4M
NI04721	Children H3-H1	4	M	Serology	2015-Nov (PCR)	1	33	3.9M	8.1M
NI01392	Children H3-H1	3	F	2014-Nov (PCR)	2015-Nov (PCR)	1	52	3.3M	3.5M
NI00498	Children H3-H1	3	M	2013-Jul (PCR)	2015-Nov (PCR)	-3	28	4.5M	9M
NI00436	Children H3-H1	2	M	Serology	2015-Nov (PCR)	1	43	2.6M	3M
NI04359	Children H3-H1	3	M	Serology	2015-Nov (PCR)	1	33	2.1M	4.5M
NI06063	Children H3-H1	3	F	Serology	2015-Nov (PCR)	0	38	1.2M	4M
NI05269	Children H3-H1	4	F	2014-Nov (PCR)	2015-Nov (PCR)	2	53	5.4M	N/A
NI02749	Children H3-H1	2	M	Serology	2015-Nov (PCR)	-1	29	2.1M	4.8M
NI06809	Children H3-H1	3	M	Serology	2015-Dec (PCR)	1	32	3.5M	9M
NI04816	Children H3-H1	3	F	Serology	2018-Nov (PCR)	3	34	4M	5.5M
NI06807	Children H3-H1	2	M	Serology	2015-Nov (PCR)	-3	28	6M	5.5M
NI03152	Children H1-H3	2	M	2019-Oct (PCR)	2018-Nov (PCR)	2	32	10M	2.7M
NI01519	Children H1-H3	2	M	2017-Sep (PCR)	2015-Dec (PCR)	1	31	6M	1.8M
NI06434	Children H1-H3	6	F	2022-Jun (PCR)	2019-Nov (PCR)	-2	28	6.5M	5.8M
NI06541	Children H1-H3	2	M	2019-Oct (PCR)	Serology	1	32	5.8M	10M
NI00526	Children H1-H3	3	M	2022-Jun (PCR)	Serology	2	30	6.4M	6.4M
NI01394	Children H1-H3	2	F	2019-Oct (PCR)	Serology	2	32	20M	5.4M
NI05446	Children H1-H3	5	M	2019-Nov (PCR)	2015-Nov (PCR)	3	33	4.5M	9M

Supplementary Table 2: Antigen probes for sorting HA-specific B cells.

Adults H1	Children H1 only	Children H3-H1	Children H1-H3
H1 A/California/7/2009	H1 A/California/7/2009	H1 A/California/7/2009	H1 A/California/7/2009
H1 A/Michigan/45/2015	H1 A/Michigan/45/2015	H1 A/Michigan/45/2015	H1 A/Michigan/45/2015
H1 A/Wisconsin/588/2019	H1 A/Brisbane/02/2018	H1 A/Wisconsin/588/2019	H1 A/Brisbane/02/2018
cH8/1 (stalk of H1 CA09)	H1 A/Wisconsin/588/2019	cH8/1 (stalk of H1 CA09)	H1 A/Wisconsin/588/2019
H3 A/Perth/16/2009	H1 A/Wisconsin/67/2022	H3 A/Perth/16/2009	H1 A/Wisconsin/67/2022
H3 A/Hong Kong/4801/2014	cH8/1 (stalk of H1 CA09)	H3 A/Hong Kong/4801/2014	cH8/1 (stalk of H1 CA09)
H3 A/Darwin/9/2021	H3 A/Hong Kong/4801/2014	H3 A/Darwin/9/2021	H3 A/Hong Kong/4801/2014
	H3 A/Darwin/9/2021		H3 A/Kansas/14/2017
	cH4/3 (stalk of H3 HK14)		H3 A/Minnesota/41/2019
			H3 A/Darwin/9/2021
			H3 A/Massachusetts/18/2022
			cH4/3 (stalk of H3 HK14)

Supplementary Table 3: Cryo-EM data collection, refinement and validation statistics for NI06063_d30_103 and NI04359_d30_240.

**Map**	**NI06063_d30_103 Fab + HK14 H3 HA**	**NI06063_d30_103 Fab + Mich15 H1 HA**	**NI04359_d30_240 Fab + HK14 H3 HA**	**NI04359_d30_240 Fab + Mich15 H1 HA**
EMDB	EMD-70233	EMD-70234	EMD-70235	EMD-70236
**Data collection**				
Microscope	TFS Glacios2	TFS Glacios	TFS Glacios2	TFS Glacios
Voltage (kV)	200	200	200	200
Detector	Falcon 4	Falcon 4	Falcon 4	Falcon 4
Recording mode	Counting	Counting	Counting	Counting
Nominal magnification	190,000x	190,000x	190,000x	190,000x
Movie micrograph pixel size (Å)	0.718	0.725	0.718	0.725
Dose rate (e^-/[(camera pixel)*s])	8.705	5.26	8.705	5.26
EER number of fractions	40	40	40	40
Movie micrograph exposure time (s)	2.67	4.48	2.67	4.48
Total dose (e^-/Å2)	45.09	44.87	45.09	44.87
Defocus range (μm)	-0.6 to -1.4	-0.7 to -1.4	-0.6 to -1.4	-0.7 to -1.4
**EM data processing**				
Number of movie micrographs	1,264	4,107	2,516	5,879
Number of particle images in map	156,709	276,374	196,828	84,345
Symmetry	C3	C3	C3	C3
Map resolution (FSC 0.143; Å)	2.62	2.96	2.65	3.12
Map sharpening B-factor ($Å^2$)	-71.4	-104.5	-59.7	-85.8
**Structure building and validation**				
Number of atoms in deposited model				
HA	11,577	11,649	11,577	11,649
Fab Fv	5,166	5,166	5,283	5,283
Glycans	759	294	768	
MolProbity score	1.11	0.95	1.05	1.10
Clashscore	1.54	0.89	2.61	1.10
Map correlation coefficient, CC (Mask)	0.79	0.81	0.74	0.79
EMRinger score	3.19	4.32	2.44	2.89
d FSC model (0.5; Å)	3.0	3.2	3.2	3.3
RMSD Bonds				
Bond length [Å]	0.005	0.004	0.007	0.005
Bond angles [˚]	0.845	0.660	1.050	0.808
Ramachandran plot				
Favored (%)	96.66	96.95	98.36	95.85
Allowed (%)	3.34	3.05	1.64	4.15
Outliers (%)	0.00	0.00	0.00	0.00
Side chain rotamer outliers (%)	0.82	0.82	0.00	0.96
Cβ outliers (%)	0.00	0.00	0.25	0.00
PDB	9O8Q	9O8R	9O8S	9O8T

Supplementary Table 4: Intermolecular contacts of NI06063_d30_103 with HK14 H3 and Mich15 H1.

HK14 H3 Chain	Amino Name 1	Amino number 1	Atom	d30_103 Chain	Amino Name 2	Amino number 2	Antibody residue origin	Distance (Å)	Predicted interaction
A	ASP	291_	OD2-NH2	H	ARG	100_A	Unknown (Non-templated)	3.17	Salt-Bridge
A	THR	318_	CB-CD2	H	LEU	99_	Germline	3.76	Van-der-Waals
B	VAL	18_	O-CD2	L	LEU	56_	Somatic mutation	3.31	Van-der-Waals
B	ASP	19_	O-CB	L	LEU	56_	Somatic mutation	3.72	Van-der-Waals
B	ASP	19_	OD1-CA	L	GLY	57_	Germline	3.49	Van-der-Waals
B	TRP	21_	CD1-CD1	L	LEU	56_	Somatic mutation	3.77	Hydr-Phbc
B	LYS	39_	CE-OE1	H	GLN	1_	Germline	3.39	Van-der-Waals
B	GLN	42_	OE1-CG2	H	VAL	2_	Germline	3.77	Van-der-Waals
B	GLN	42_	CG-O	H	GLY	26_	Germline	3.7	Van-der-Waals
B	GLN	42_	NE2-OH	H	TYR	32_	Germline	3.22	Hydro-Bond
B	ILE	45_	CG2-OH	H	TYR	32_	Germline	3.4	Van-der-Waals
B	ILE	45_	CG2-CD1	H	LEU	99_	Germline	3.98	Hydr-Phbc
B	ASP	46_	OD2-CA	H	PHE	27_	Germline	3.21	Van-der-Waals
B	ASP	46_	OD2-N	H	THR	28_	Germline	2.54	Hydro-Bond
B	ASP	46_	OD2-OG1	H	THR	28_	Germline	3.21	Hydro-Bond
B	ASP	46_	O-ND2	H	ASN	31_	Somatic mutation	2.74	Hydro-Bond
B	ASP	46_	OD1-OH	H	TYR	32_	Germline	2.53	Hydro-Bond
B	ASN	49_	ND2-OH	H	TYR	32_	Germline	3	Hydro-Bond
B	ASN	49_	OD1-CB	H	GLN	97_	Germline	3.39	Van-der-Waals
B	ASN	49_	OD1-N	H	TRP	98_	Germline	3.2	Hydro-Bond
B	GLY	50_	CA-OD1	H	ASN	31_	Somatic mutation	3.62	Van-der-Waals
B	LEU	52_	CB-CD1	H	TRP	98_	Germline	3.49	Hydr-Phbc
B	LEU	52_	CD1-CB	H	TRP	98_	Germline	3.83	Hydr-Phbc
B	LEU	52_	CB-CB	H	TRP	98_	Germline	3.9	Hydr-Phbc
B	LEU	52_	CB-CG	H	TRP	98_	Germline	3.93	Hydr-Phbc
B	LEU	52_	CD1-CD2	H	LEU	99_	Germline	3.47	Hydr-Phbc
B	ASN	53_	ND2-O	H	ASN	31_	Somatic mutation	2.9	Hydro-Bond
B	ASN	53_	ND2-CD2	H	TYR	52_A	Germline	3.57	Van-der-Waals
B	ASN	53_	OD1-NE2	H	GLN	97_	Germline	2.43	Hydro-Bond
B	ASN	53_	OD1-NE1	H	TRP	98_	Germline	2.67	Hydro-Bond
B	ILE	56_	CG2-NH2	H	ARG	100_A	Unknown (Non-templated)	3.49	Van-der-Waals
B	ILE	56_	CG2-CZ3	H	TRP	98_	Germline	3.4	Hydr-Phbc
B	ILE	56_	CG2-CE3	H	TRP	98_	Germline	3.44	Hydr-Phbc
B	ILE	56_	CG2-CH2	H	TRP	98_	Germline	3.68	Hydr-Phbc
B	ILE	56_	CG2-CD2	H	TRP	98_	Germline	3.75	Hydr-Phbc
B	ILE	56_	CG2-CZ2	H	TRP	98_	Germline	3.98	Hydr-Phbc
B	ILE	56_	CG2-CE2	H	TRP	98_	Germline	3.99	Hydr-Phbc
B	ILE	56_	CB-CD2	H	TRP	98_	Germline	3.99	Hydr-Phbc
B	LYS	58_	NZ-OH	H	TYR	52_A	Germline	2.83	Hydro-Bond
B	LYS	58_	NZ-OD2	H	ASP	53_	Germline	3.3	Salt-Bridge
C	THR	30_	O-OD1	H	ASN	31_	Somatic mutation	3.73	Van-der-Waals
C	ASN	31_	ND2-CG2	H	THR	28_	Germline	3.6	Van-der-Waals
C	ASP	32_	OD1-CB	H	SER	30_	Germline	3.25	Van-der-Waals
C	ASP	32_	OD2-CE2	H	TYR	52_A	Germline	2.97	Van-der-Waals
C	ARG	33_	CB-OG	H	SER	30_	Germline	3.32	Van-der-Waals
C	ARG	33_	NE-CZ	H	TYR	73_	Somatic mutation	3.6	Van-der-Waals

Mich15 H1 Chain	Amino Name 1	Amino number 1	Atom	d30_103 Chain	Amino Name 2	Amino number 2	Antibody residue origin	Distance (Å)	Predicted interaction
A	HIS	18_	NE2-CD2	L	LEU	56_	Somatic mutation	3.72	Van-der-Waals
A	SER	291_	OG-CH2	H	TRP	98_	Germline	3.57	Van-der-Waals
A	LEU	292_	CD2-NH1	H	ARG	100_A	Unknown (Non-templated)	3.69	Van-der-Waals
A	LEU	292_	CD2-CZ3	H	TRP	98_	Germline	3.82	Hydr-Phbc
A	THR	318_	OG1-CD2	H	LEU	99_	Germline	3.23	Van-der-Waals
A	VAL	40_	CG2-O	H	TRP	98_	Germline	3.44	Van-der-Waals
A	VAL	40_	CG1-CE3	H	TRP	98_	Germline	3.92	Hydr-Phbc
B	VAL	18_	O-CD2	L	LEU	56_	Somatic mutation	3.02	Van-der-Waals
B	ASP	19_	C-CB	L	LEU	56_	Somatic mutation	3.81	Van-der-Waals
B	GLY	20_	CA-CD1	L	LEU	56_	Somatic mutation	3.98	Hydr-Phbc
B	TRP	21_	CD1-CD1	L	LEU	56_	Somatic mutation	3.67	Hydr-Phbc
B	VAL	18_	O-N	L	GLY	57_	Germline	2.75	Hydro-Bond
B	TRP	21_	CZ2-CD1	H	LEU	99_	Germline	3.34	Hydr-Phbc
B	TRP	21_	CZ2-CD2	H	LEU	99_	Germline	3.64	Hydr-Phbc
B	TRP	21_	CH2-CD1	H	LEU	99_	Germline	3.7	Hydr-Phbc
B	TRP	21_	CH2-CD2	H	LEU	99_	Germline	3.97	Hydr-Phbc
B	ASP	19_	CA-N	L	GLY	57_	Germline	3.65	Van-der-Waals
B	LEU	38_	CB-CG	H	GLN	1_	Germline	3.62	Van-der-Waals
B	LYS	39_	NZ-OE1	H	GLN	1_	Germline	2.54	Hydro-Bond
B	GLN	42_	OE1-CG2	H	VAL	2_	Germline	3.8	Van-der-Waals
B	GLN	42_	CG-O	H	GLY	26_	Germline	3.47	Van-der-Waals
B	GLN	42_	NE2-CB	H	PHE	27_	Germline	3.59	Van-der-Waals
B	GLN	42_	NE2-OH	H	TYR	32_	Germline	3.41	Hydro-Bond
B	ILE	45_	CG2-OH	H	TYR	32_	Germline	3.4	Van-der-Waals
B	ILE	45_	CG2-CD1	H	LEU	99_	Germline	3.57	Hydr-Phbc
B	ILE	45_	CG1-CD1	H	LEU	99_	Germline	3.67	Hydr-Phbc
B	ASP	46_	OD2-CA	H	PHE	27_	Germline	3.51	Van-der-Waals
B	ASP	46_	OD2-N	H	THR	28_	Germline	2.71	Hydro-Bond
B	ASP	46_	OD2-OG1	H	THR	28_	Germline	2.8	Hydro-Bond
B	ASP	46_	O-ND2	H	ASN	31_	Somatic mutation	2.93	Hydro-Bond
B	ASP	46_	OD1-OH	H	TYR	32_	Germline	2.44	Hydro-Bond
B	THR	49_	CG2-O	H	ASN	31_	Somatic mutation	3.98	Van-der-Waals
B	THR	49_	CG2-CB	H	GLN	97_	Germline	3.55	Van-der-Waals
B	ASN	50_	OD1-OD1	H	ASN	31_	Somatic mutation	3.18	Van-der-Waals
B	VAL	52_	CG1-CD1	H	TRP	98_	Germline	3.44	Hydr-Phbc
B	VAL	52_	CG1-CG	H	TRP	98_	Germline	3.47	Hydr-Phbc
B	VAL	52_	CG1-CD2	H	TRP	98_	Germline	3.59	Hydr-Phbc
B	VAL	52_	CG1-CE2	H	TRP	98_	Germline	3.63	Hydr-Phbc
B	VAL	52_	CB-CD1	H	TRP	98_	Germline	3.73	Hydr-Phbc
B	ASN	53_	ND2-O	H	ASN	31_	Somatic mutation	3.22	Hydro-Bond
B	ASN	53_	O-OH	H	TYR	52_A	Germline	3.35	Hydro-Bond
B	ASN	53_	CB-NE1	H	TRP	98_	Germline	3.8	Van-der-Waals
B	ILE	56_	CD1-CZ2	H	TRP	98_	Germline	3.28	Hydr-Phbc
B	ILE	56_	CD1-CH2	H	TRP	98_	Germline	3.65	Hydr-Phbc
B	ILE	56_	CD1-CE2	H	TRP	98_	Germline	3.87	Hydr-Phbc
B	GLU	57_	CB-OH	H	TYR	52_A	Germline	3.53	Van-der-Waals
B	GLU	57_	OE2-OD1	H	ASP	53_	Germline	3.88	Van-der-Waals

Supplementary Table 5: Intermolecular contacts of NI04359_d30_240 with HK14 H3 and Mich15 H1.

HK14 H3 Chain	Amino Name 1	Amino number 1	Atom	d30_240 Chain	Amino Name 2	Amino number 2	Antibody residue origin	Distance (Å)	Predicted interaction
B	ASP	19	OD2-CA	H	GLY	55	Germline	3.44	Van-der-Waals
B	GLY	20	CA-OH	H	TYR	100_H	Germline	3.93	Van-der-Waals
B	TRP	21	CZ2-CD1	H	ILE	100_E	Unknown (Non-templated)	3.88	Hydr-Phbc
B	LEU	38	CD1-OG	H	SER	52	Germline	3.85	Van-der-Waals
B	LEU	38	CD1-OH	H	TYR	58	Germline	3.32	Van-der-Waals
B	LEU	38	CD1-CB	H	TYR	100_H	Germline	3.78	Hydr-Phbc
B	LEU	38	CD2-CB	H	TYR	100_H	Germline	3.97	Hydr-Phbc
B	LEU	38	CD2-CD1	H	TYR	100_H	Germline	3.71	Hydr-Phbc
B	LEU	38	CD2-CG	H	TYR	100_H	Germline	3.73	Hydr-Phbc
B	LEU	38	CB-CE2	H	TYR	100_I	Germline	3.69	Hydr-Phbc
B	LEU	38	CD1-CD2	H	TYR	100_I	Germline	3.4	Hydr-Phbc
B	LEU	38	CD1-CE2	H	TYR	100_I	Germline	3.54	Hydr-Phbc
B	LYS	39	CA-OH	H	TYR	100_J	Germline	3.47	Van-der-Waals
B	GLN	42	CD-O	H	GLY	97	Unknown (Non-templated)	3.9	Van-der-Waals
B	GLN	42	NE2-O	H	GLY	97	Unknown (Non-templated)	2.97	Hydro-Bond
B	GLN	42	CD-CA	H	GLY	100_G	Unknown (Non-templated)	3.93	Van-der-Waals
B	GLN	42	OE1-N	H	TYR	100_H	Germline	2.81	Hydro-Bond
B	GLN	42	CB-CD2	H	TYR	100_J	Germline	3.86	Van-der-Waals
B	ILE	45	CA-CD1	H	ILE	100_E	Unknown (Non-templated)	3.72	Hydr-Phbc
B	ILE	45	CB-CD1	H	ILE	100_E	Unknown (Non-templated)	3.71	Hydr-Phbc
B	ILE	45	CG1-CD1	H	ILE	100_E	Unknown (Non-templated)	3.51	Hydr-Phbc
B	ILE	45	CG2-CD1	H	ILE	100_E	Unknown (Non-templated)	3.57	Hydr-Phbc
B	ILE	45	CD1-O	H	VAL	100_F	Unknown (Non-templated)	3.71	Van-der-Waals
B	ILE	45	CG2-CA	H	GLY	100_G	Unknown (Non-templated)	3.99	Hydr-Phbc
B	ILE	45	CD1-CE1	H	TYR	100_H	Germline	3.41	Hydr-Phbc
B	ASP	46	OD1-CA	H	CYS	98	Germline	3.96	Van-der-Waals
B	ASP	46	CB-CD	H	ARG	99	Somatic mutation	3.95	Van-der-Waals
B	ASP	46	O-NH1	H	ARG	99	Somatic mutation	3.49	Hydro-Bond
B	ASP	46	OD2-OH	H	TYR	100_K	Germline	2.56	Hydro-Bond
B	ASP	46	CG-CE2	H	TYR	100_K	Germline	3.73	Van-der-Waals
B	ASN	49	ND2-CB	H	CYS	98	Germline	3.9	Van-der-Waals
B	ASN	49	ND2-SG	H	CYS	98	Germline	3.21	Hydro-Bond
B	ASN	49	CB-OG	H	SER	100	Germline	3.78	Van-der-Waals
B	ASN	49	OD1-C	H	TYR	100_D	Germline	3.92	Van-der-Waals
B	ASN	49	CG-CG1	H	ILE	100_E	Unknown (Non-templated)	3.96	Van-der-Waals
B	ASN	49	ND2-O	H	ILE	100_E	Unknown (Non-templated)	2.66	Hydro-Bond
B	ASN	49	OD1-N	H	ILE	100_E	Unknown (Non-templated)	3.14	Hydro-Bond
B	LEU	52	C-CE2	H	TYR	100_D	Germline	3.86	Van-der-Waals
B	LEU	52	CB-CD2	H	TYR	100_D	Germline	3.7	Hydr-Phbc
B	LEU	52	CD1-CB	H	TYR	100_D	Germline	3.75	Hydr-Phbc
B	ASN	53	CG-CB	H	SER	100	Germline	3.68	Van-der-Waals
B	ASN	53	ND2-OG	H	SER	100	Germline	2.61	Hydro-Bond
B	ASN	53	OD1-OG	H	SER	100	Germline	3.09	Hydro-Bond
B	ASN	53	CG-N	H	ASN	100_B	Somatic mutation	3.89	Van-der-Waals
B	ASN	53	ND2-O	H	ASN	100_B	Somatic mutation	2.99	Hydro-Bond
B	ASN	53	OD1-N	H	ASN	100_B	Somatic mutation	2.95	Hydro-Bond
B	ASN	53	CA-CE2	H	TYR	100_D	Germline	3.67	Van-der-Waals
B	ILE	56	C-OH	H	TYR	100_D	Germline	3.57	Van-der-Waals
B	ILE	56	CB-CE1	H	TYR	100_D	Germline	3.86	Hydr-Phbc
B	ILE	56	CB-CZ	H	TYR	100_D	Germline	3.68	Hydr-Phbc
B	ILE	56	CD1-CE1	H	TYR	100_D	Germline	4	Hydr-Phbc
B	ILE	56	CG2-CE1	H	TYR	100_D	Germline	3.33	Hydr-Phbc
B	ILE	56	CG2-CZ	H	TYR	100_D	Germline	3.48	Hydr-Phbc
B	ILE	56	O-OH	H	TYR	100_D	Germline	3.09	Hydro-Bond
C	THR	30	C-NH2	H	ARG	99	Somatic mutation	3.72	Van-der-Waals
C	THR	30	O-NH1	H	ARG	99	Somatic mutation	3.23	Hydro-Bond
C	THR	30	O-NH2	H	ARG	99	Somatic mutation	3.22	Hydro-Bond
C	ASN	31	CA-CZ	H	ARG	99	Somatic mutation	3.96	Van-der-Waals

Mich15 H1 Chain	Amino Name 1	Amino number 1	Atom	d30_240 Chain	Amino Name 2	Amino number 2	Antibody residue origin	Distance (Å)	Predicted interaction
A	THR	318_	CB-CD1	H	ILE	100_E	Unknown (Non-templated)	3.92	Van-der-Waals
A	VAL	40_	CG2-CB	H	TYR	100_D	Germline	3.52	Hydr-Phbc
B	VAL	18_	CG2-CB	H	SER	53_	Germline	3.31	Van-der-Waals
B	ASP	19_	O-OH	H	TYR	100_H	Germline	3.46	Hydro-Bond
B	ASP	19_	O-CE2	H	TYR	100_H	Germline	3.13	Van-der-Waals
B	ASP	19_	CB-OG	H	SER	53_	Germline	3.73	Van-der-Waals
B	ASP	19_	OD2-OG1	H	THR	56_	Somatic mutation	2.59	Hydro-Bond
B	GLY	20_	CA-OH	H	TYR	100_H	Germline	3.92	Van-der-Waals
B	TRP	21_	CZ2-CG2	H	ILE	100_E	Unknown (Non-templated)	3.57	Hydr-Phbc
B	TRP	21_	CH2-CD1	H	ILE	100_E	Unknown (Non-templated)	3.83	Hydr-Phbc
B	TRP	21_	CH2-CG2	H	ILE	100_E	Unknown (Non-templated)	3.9	Hydr-Phbc
B	LEU	38_	CG-CE1	H	TYR	100_H	Germline	3.71	Hydr-Phbc
B	LEU	38_	CD2-CE2	H	TYR	100_I	Germline	3.43	Hydr-Phbc
B	LEU	38_	CD2-CZ	H	TYR	100_I	Germline	3.54	Hydr-Phbc
B	LEU	38_	CD2-OH	H	TYR	58_	Germline	3.63	Van-der-Waals
B	LYS	39_	CA-OH	H	TYR	100_J	Germline	3.93	Van-der-Waals
B	THR	41_	CG2-OH	H	TYR	100_H	Germline	3.06	Van-der-Waals
B	GLN	42_	OE1-CA	H	GLY	100_G	Unknown (Non-templated)	3.25	Van-der-Waals
B	GLN	42_	OE1-N	H	TYR	100_H	Germline	3.16	Hydro-Bond
B	ILE	45_	CG2-CG2	H	ILE	100_E	Unknown (Non-templated)	3.81	Hydr-Phbc
B	ILE	45_	CG1-CG2	H	ILE	100_E	Unknown (Non-templated)	3.82	Hydr-Phbc
B	ILE	45_	CG2-CB	H	ILE	100_E	Unknown (Non-templated)	3.95	Hydr-Phbc
B	ILE	45_	CD1-O	H	VAL	100_F	Unknown (Non-templated)	3.48	Van-der-Waals
B	ASP	46_	OD2-OH	H	TYR	100_K	Germline	2.59	Hydro-Bond
B	ASP	46_	OD1-CB	H	ARG	99_	Somatic mutation	3.23	Van-der-Waals
B	ILE	48_	CG2-CD1	H	ILE	100_E	Unknown (Non-templated)	3.7	Hydr-Phbc
B	THR	49_	CG2-OG	H	SER	100_	Germline	3.36	Van-der-Waals
B	THR	49_	OG1-CD1	H	ILE	100_E	Unknown (Non-templated)	3.49	Van-der-Waals
B	VAL	52_	CG1-CE2	H	TYR	100_D	Germline	3.46	Hydr-Phbc
B	VAL	52_	CG1-CD2	H	TYR	100_D	Germline	3.54	Hydr-Phbc
B	ASN	53_	ND2-CB	H	SER	100_	Germline	3.29	Van-der-Waals
B	ASN	53_	ND2-OG	H	SER	100_	Germline	3.34	Hydro-Bond
B	ASN	53_	OD1-OH	H	TYR	100_D	Germline	3.07	Hydro-Bond
B	ILE	56_	CD1-OH	H	TYR	100_D	Germline	2.96	Van-der-Waals
B	ILE	56_	CD1-CZ	H	TYR	100_D	Germline	3.34	Hydr-Phbc
B	ILE	56_	CD1-CE2	H	TYR	100_D	Germline	3.84	Hydr-Phbc
B	ILE	56_	CD1-CE1	H	TYR	100_D	Germline	3.96	Hydr-Phbc

Supplementary Table 6: Recombinant HA proteins used in indicated assays.

Name	Strain	Subtype	Assay
SC18	A/South Carolina/1/1918	H1	High avidity ELISA
Den57	A/Denver/1/1957	H1	High avidity ELISA
USSR77	A/USSR/90/1977	H1	High avidity ELISA
NC99	A/New Caledonia/20/1999	H1	High avidity ELISA; Molecular Dynamics simulation
SI06	A/Solomon Islands/3/2006	H1	Cryo-EM
Bris07	A/Brisbane/59/2007	H1	High avidity ELISA
Cal09	A/California/7/2009	H1	High avidity ELISA
Mich15	A/Michigan/45/2015	H1	Serum ELISA; High avidity ELISA; Cryo-EM
Bris18	A/Brisbane/02/2018	H1	High avidity ELISA
Wis19	A/Wisconsin/588/2019	H1	High avidity ELISA
Wis22	A/Wisconsin/67/2022	H1	High avidity ELISA
Sing57	A/Singapore/1/1957	H2	High avidity ELISA
Ghana21	A/chicken/Ghana/AVL-763_21VIR7050-39/2021	H5	High avidity ELISA
AH18	A/Anhui-Lujiang/39/2018	H9	High avidity ELISA
HK68	A/Hongkong/1/1968	H3	High avidity ELISA
NY95	A/New York/680/1995	H3	High avidity ELISA
Wis05	A/Wisconsin/67/2005	H3	High avidity ELISA
Perth09	A/Perth/16/2009	H3	High avidity ELISA
Vic11	A/Victoria/361/2011	H3	High avidity ELISA
HK14	A/Hongkong/4801/2014	H3	Serum ELISA; High avidity ELISA; Cryo-EM
Kan17	A/Kansas/14/2017	H3	High avidity ELISA
Dar21	A/Darwin/9/2021	H3	High avidity ELISA
SH13	A/Shanghai/02/2013	H7	High avidity ELISA
JX13	A/Jiangxi-Donghu/346/2013	H10	High avidity ELISA
MA82	A/mallard/Astrakhan/263/1982	H14	High avidity ELISA
cH8/1	H8 head: A/mallard/Sweden/24/2002<br>H1 stalk: A/California/04/2009	Chimeric	High avidity ELISA
cH4/3	H4 head: A/duck/Czechoslovakia/1956<br>H3 stalk: A/Hongkong/4801/2014	Chimeric	High avidity ELISA

Supplementary Table 7: Influenza viruses used in indicated assays.

Name	Strain	Subtype	Assay
PR34	A/Puerto Rico/8/1934	H1N1	FRNT
Braz78	A/Brazil/11/1978	H1N1	FRNT
NC99	A/New Caledonia/20/1999	H1N1	FRNT
KA01	A/Kawasaki/173/2001	H1N1	MN
BR07	A/Brisbane/59/2007	H1N1	FRNT; MN
Cal09	A/California/7/2009	H1N1	FRNT
Mich15	A/Michigan/45/2015	H1N1	FRNT
Wis19	A/Wisconsin/588/2019	H1N1	FRNT
NL99	A/white fronted goose/Netherlands/22/1999	H2N2	MN
HK68	A/Hongkong/1/1968	H3N2	FRNT
Wis05	A/Wisconsin/67/2005	H3N2	FRNT
PE09	A/Perth/16/2009	H3N2	MN
Vic11	A/Victoria/361/2011	H3N2	FRNT; MN
TX12	A/Texas/50/2012	H3N2	MN
HK14	A/Hongkong/4801/2014	H3N2	FRNT; MN
SI16	A/Singapore/Infimh-16-0019/2016	H3N2	MN
NV16	A/Nevada/22/2016	H3N2	MN
KA17	A/Kansas/14/2017	H3N2	MN
SW17	A/Switzerland/8060/2017	H3N2	MN
HK19	A/Hong Kong/45/2019	H3N2	MN
TK19	A/Tokyo/UT-GR117-0/2019	H3N2	MN
CB20	A/Cambodia/e0826360/2020	H3N2	MN
Dar21	A/Darwin/6/2021	H3N2	FRNT; MN
CA23	A/California/45/2023	H3N2	MN
WI83	A/Blue-winged teal/Wisconsin/402/1983	H4N6	MN
SC14	A/Sichuan/26221/2014_PR8(6)_IDCDC-RG42A	H5N6	MN
DB09	A/shorebird/Delaware Bay/230/2009	H6N1	MN
TX79	A/turkey/Texas/48/1979	H7N2	MN
ON67	A/turkey/Ontario/6118/1967	H8N4	MN
WI66	A/turkey/Wisconsin/1/1966	H9N2	MN
DB09	A/shorebird/Delaware Bay/338/2009	H10N1	MN
MS10	A/American green-winged teal/Mississippi/300/2010	H11N9	MN
WI09	A/mallard/Wisconsin/4216/2009	H12N5	MN

Supplementary Table 8: PCR primers for deep mutational scanning.

Primer ID	Sequence	Primer ID	Sequence	Primer ID	Sequence
Cassette1_1	GGAAATCCAGAGTGTGAATCANNKTCTACCGCAAGTTCATGGTCCTACATTGTGGAAACA	Cassette8_8	AAGACAAGTTCATGGCCCAATCATGACTCTAACAAGGGGGTANNKGCAGCATGTCCTCAC	Cassette16_7	TCTACTACTGCTGACCAACAAAGCCTCTATCAAAACGCCNNKGCATATGTTTTTGTGGGG
Cassette1_2	GGAAATCCAGAGTGTGAATCACTTNNKACAGCCAGTTCATGGTCCTACATTGTGGAAACA	Cassette9_1	GACTCGAACAAAGGTGTAACGNNKGCCTGCCCTCACGCTGGAGCAAAAAGCTTCTACAAA	Cassette16_8	TCTACTACTGCTGACCAACAAAGCCTCTATCAGAATGCCGATNNKTATGTTTTTGTGGGG
Cassette1_3	GGAAATCCAGAGTGTGAATCACTCTCTNNKGCCAGCTCATGGTCCTACATTGTGGAAACA	Cassette9_2	GACTCGAACAAAGGTGTAACGGCCNNKTGTCCGCACGCTGGAGCAAAAAGCTTCTACAAA	Cassette17_1	CTCTATCAGAATGCAGATGCANNKGTATTCGTGGGGACATCAAGATACAGCAAGAAGTTC
Cassette1_4	GGAAATCCAGAGTGTGAATCACTTTCCACCNNKAGCTCATGGTCCTACATTGTGGAAACA	Cassette9_3	GACTCGAACAAAGGTGTAACGGCAGCANNKCCGCATGCTGGAGCAAAAAGCTTCTACAAA	Cassette17_2	CTCTATCAGAATGCAGATGCATACNNKTTTGTAGGGACATCAAGATACAGCAAGAAGTTC
Cassette1_5	GGAAATCCAGAGTGTGAATCACTCTCTACAGCANNKTCTTGGTCCTACATTGTGGAAACA	Cassette9_4	GACTCGAACAAAGGTGTAACGGCAGCATGCNNKCACGCAGGAGCAAAAAGCTTCTACAAA	Cassette17_3	CTCTATCAGAATGCAGATGCATATGTANNKGTAGGTACATCAAGATACAGCAAGAAGTTC
Cassette1_6	GGAAATCCAGAGTGTGAATCACTCTCCACCGCCAGTNNKTGGTCCTACATTGTGGAAACA	Cassette9_5	GACTCGAACAAAGGTGTAACGGCAGCCTGTCCTNNKGCAGGAGCAAAAAGCTTCTACAAA	Cassette17_4	CTCTATCAGAATGCAGATGCATACGTTTTCNNKGGTACATCAAGATACAGCAAGAAGTTC
Cassette1_7	GGAAATCCAGAGTGTGAATCACTTTCTACAGCAAGCTCANNKTCCTACATTGTGGAAACA	Cassette9_6	GACTCGAACAAAGGTGTAACGGCCGCATGTCCTCATNNKGGAGCAAAAAGCTTCTACAAA	Cassette17_5	CTCTATCAGAATGCAGATGCATATGTATTTGTGNNKACCTCAAGATACAGCAAGAAGTTC
Cassette1_8	GGAAATCCAGAGTGTGAATCACTCTCCACCGCAAGCTCTTGGNNKTACATTGTGGAAACA	Cassette9_7	GACTCGAACAAAGGTGTAACGGCCGCCTGCCCGCATGCTNNKGCAAAAAGCTTCTACAAA	Cassette17_6	CTCTATCAGAATGCAGATGCATATGTTTTCGTAGGGNNKTCAAGATACAGCAAGAAGTTC
Cassette2_1	TCCACAGCAAGTTCATGGTCCNNKATCGTAGAAACATCTAATTCAGACAATGGAACGTGT	Cassette9_8	GACTCGAACAAAGGTGTAACGGCAGCCTGCCCGCATGCAGGANNKAAAAGCTTCTACAAA	Cassette17_7	CTCTATCAGAATGCAGATGCATACGTATTTGTGGGTACANNKAGATACAGCAAGAAGTTC
Cassette2_2	TCCACAGCAAGTTCATGGTCCTATNNKGTGGAGACATCTAATTCAGACAATGGAACGTGT	Cassette10_1	GCATGTCCTCACGCTGGAGCANNKAGTTTTTACAAAAACTTGATATGGCTAGTTAAAAAA	Cassette17_8	CTCTATCAGAATGCAGATGCATATGTTTTTGTAGGTACCTCANNKTACAGCAAGAAGTTC
Cassette2_3	TCCACAGCAAGTTCATGGTCCTATATTNNKGAAACCTCTAATTCAGACAATGGAACGTGT	Cassette10_2	GCATGTCCTCACGCTGGAGCAAAGNNKTTCTATAAAAACTTGATATGGCTAGTTAAAAAA	Cassette18_1	GTTTTTGTGGGGACATCAAGANNKAGTAAAAAGTTCAAGCCGGAAATAGCAACAAGACCC
Cassette2_4	TCCACAGCAAGTTCATGGTCCTACATCGTGNNKACCTCTAATTCAGACAATGGAACGTGT	Cassette10_3	GCATGTCCTCACGCTGGAGCAAAGAGCNNKTACAAGAACTTGATATGGCTAGTTAAAAAA	Cassette18_2	GTTTTTGTGGGGACATCAAGATATNNKAAGAAATTCAAGCCGGAAATAGCAACAAGACCC
Cassette2_5	TCCACAGCAAGTTCATGGTCCTACATTGTAGAGNNKTCTAATTCAGACAATGGAACGTGT	Cassette10_4	GCATGTCCTCACGCTGGAGCAAAAAGTTTCNNKAAGAACTTGATATGGCTAGTTAAAAAA	Cassette18_3	GTTTTTGTGGGGACATCAAGATATAGCNNKAAGTTTAAGCCGGAAATAGCAACAAGACCC
Cassette2_6	TCCACAGCAAGTTCATGGTCCTATATCGTAGAGACCNNKAATTCAGACAATGGAACGTGT	Cassette10_5	GCATGTCCTCACGCTGGAGCAAAAAGCTTTTATNNKAACTTGATATGGCTAGTTAAAAAA	Cassette18_4	GTTTTTGTGGGGACATCAAGATACAGTAAGNNKTTTAAGCCGGAAATAGCAACAAGACCC
Cassette2_7	TCCACAGCAAGTTCATGGTCCTATATCGTGGAAACCTCGNNKTCAGACAATGGAACGTGT	Cassette10_6	GCATGTCCTCACGCTGGAGCAAAGAGTTTTTATAAGNNKTTGATATGGCTAGTTAAAAAA	Cassette18_5	GTTTTTGTGGGGACATCAAGATACAGCAAAAAANNKAAGCCGGAAATAGCAACAAGACCC
Cassette2_8	TCCACAGCAAGTTCATGGTCCTATATTGTAGAGACATCGAATNNKGACAATGGAACGTGT	Cassette10_7	GCATGTCCTCACGCTGGAGCAAAGAGCTTTTATAAAAATNNKATATGGCTAGTTAAAAAA	Cassette18_6	GTTTTTGTGGGGACATCAAGATATAGTAAAAAATTTNNKCCGGAAATAGCAACAAGACCC
Cassette3_1	ATTGTGGAAACATCTAATTCANNKAACGGTACGTGTTACCCAGGAGATTTCATCAATTAT	Cassette10_8	GCATGTCCTCACGCTGGAGCAAAGAGCTTCTATAAGAATTTGNNKTGGCTAGTTAAAAAA	Cassette18_7	GTTTTTGTGGGGACATCAAGATATAGCAAGAAGTTCAAANNKGAAATAGCAACAAGACCC
Cassette3_2	ATTGTGGAAACATCTAATTCAGATNNKGGAACCTGTTACCCAGGAGATTTCATCAATTAT	Cassette11_1	AGCTTCTACAAAAACTTGATANNKCTCGTAAAAAAAGGAAATTCATACCCAAAGCTTAAC	Cassette18_8	GTTTTTGTGGGGACATCAAGATATAGCAAGAAATTTAAACCGNNKATAGCAACAAGACCC
Cassette3_3	ATTGTGGAAACATCTAATTCAGATAATNNKACGTGCTACCCAGGAGATTTCATCAATTAT	Cassette11_2	AGCTTCTACAAAAACTTGATATGGNNKGTAAAGAAGGGAAATTCATACCCAAAGCTTAAC	Cassette19_1	AGCAAGAAGTTCAAGCCGGAANNKGCCACCAGACCCAAAGTGAGGGATCAAGAAGGGAGA
Cassette3_4	ATTGTGGAAACATCTAATTCAGACAACGGANNKTGCTACCCAGGAGATTTCATCAATTAT	Cassette11_3	AGCTTCTACAAAAACTTGATATGGCTCNNKAAGAAAGGTAATTCATACCCAAAGCTTAAC	Cassette19_2	AGCAAGAAGTTCAAGCCGGAAATCNNKACAAGGCCCAAAGTGAGGGATCAAGAAGGGAGA
Cassette3_5	ATTGTGGAAACATCTAATTCAGACAATGGTACCNNKTACCCAGGAGATTTCATCAATTAT	Cassette11_4	AGCTTCTACAAAAACTTGATATGGCTAGTTNNKAAGGGTAATTCATACCCAAAGCTTAAC	Cassette19_3	AGCAAGAAGTTCAAGCCGGAAATCGCANNKAGACCTAAAGTGAGGGATCAAGAAGGGAGA
Cassette3_6	ATTGTGGAAACATCTAATTCAGATAACGGTACCTGCNNKCCAGGAGATTTCATCAATTAT	Cassette11_5	AGCTTCTACAAAAACTTGATATGGCTCGTAAAANNKGGTAACTCATACCCAAAGCTTAAC	Cassette19_4	AGCAAGAAGTTCAAGCCGGAAATAGCCACANNKCCTAAAGTGAGGGATCAAGAAGGGAGA
Cassette3_7	ATTGTGGAAACATCTAATTCAGATAACGGAACGTGTTATNNKGGAGATTTCATCAATTAT	Cassette11_6	AGCTTCTACAAAAACTTGATATGGCTCGTTAAGAAGNNKAACTCATACCCAAAGCTTAAC	Cassette19_5	AGCAAGAAGTTCAAGCCGGAAATAGCAACCAGGNNKAAAGTGAGGGATCAAGAAGGGAGA
Cassette3_8	ATTGTGGAAACATCTAATTCAGATAATGGTACCTGTTATCCANNKGATTTCATCAATTAT	Cassette11_7	AGCTTCTACAAAAACTTGATATGGCTCGTAAAGAAGGGTNNKTCTTACCCAAAGCTTAAC	Cassette19_6	AGCAAGAAGTTCAAGCCGGAAATCGCCACCAGGCCTNNKGTGAGGGATCAAGAAGGGAGA
Cassette4_1	AATGGAACGTGTTACCCAGGANNKTTTATAAATTATGAGGAGCTAAGAGAGCAATTGAGC	Cassette11_8	AGCTTCTACAAAAACTTGATATGGCTCGTTAAAAAGGGAAATNNKTACCCAAAGCTTAAC	Cassette19_7	AGCAAGAAGTTCAAGCCGGAAATCGCAACCAGGCCCAAGNNKAGGGATCAAGAAGGGAGA
Cassette4_2	AATGGAACGTGTTACCCAGGAGACNNKATCAACTATGAGGAGCTAAGAGAGCAATTGAGC	Cassette12_1	CTAGTTAAAAAAGGAAATTCANNKCCTAAACTTAACCAATCCTACATTAATGATAAAGGG	Cassette19_8	AGCAAGAAGTTCAAGCCGGAAATCGCAACAAGGCCTAAGGTGNNKGATCAAGAAGGGAGA
Cassette4_3	AATGGAACGTGTTACCCAGGAGATTTTNNKAACTACGAGGAGCTAAGAGAGCAATTGAGC	Cassette12_2	CTAGTTAAAAAAGGAAATTCATATNNKAAGCTCAACCAATCCTACATTAATGATAAAGGG	Cassette20_1	GCAACAAGACCCAAAGTGAGGNNKCAGGAGGGGAGAATGAACTATTACTGGACACTAGTA
Cassette4_4	AATGGAACGTGTTACCCAGGAGACTTCATANNKTACGAGGAGCTAAGAGAGCAATTGAGC	Cassette12_3	CTAGTTAAAAAAGGAAATTCATATCCANNKCTTAATCAATCCTACATTAATGATAAAGGG	Cassette20_2	GCAACAAGACCCAAAGTGAGGGACNNKGAAGGTAGAATGAACTATTACTGGACACTAGTA
Cassette4_5	AATGGAACGTGTTACCCAGGAGATTTTATCAATNNKGAAGAGCTAAGAGAGCAATTGAGC	Cassette12_4	CTAGTTAAAAAAGGAAATTCATACCCTAAGNNKAATCAATCCTACATTAATGATAAAGGG	Cassette20_3	GCAACAAGACCCAAAGTGAGGGATCAANNKGGTAGGATGAACTATTACTGGACACTAGTA
Cassette4_6	AATGGAACGTGTTACCCAGGAGATTTCATAAACTATNNKGAGCTAAGAGAGCAATTGAGC	Cassette12_5	CTAGTTAAAAAAGGAAATTCATACCCAAAACTCNNKCAATCCTACATTAATGATAAAGGG	Cassette20_4	GCAACAAGACCCAAAGTGAGGGATCAAGAGNNKAGAATGAATTATTACTGGACACTAGTA
Cassette4_7	AATGGAACGTGTTACCCAGGAGACTTTATCAATTACGAGNNKCTAAGAGAGCAATTGAGC	Cassette12_6	CTAGTTAAAAAAGGAAATTCATATCCTAAACTCAATNNKTCCTACATTAATGATAAAGGG	Cassette20_5	GCAACAAGACCCAAAGTGAGGGATCAGGAAGGTNNKATGAATTATTACTGGACACTAGTA
Cassette4_8	AATGGAACGTGTTACCCAGGAGATTTCATCAACTACGAAGAGNNKAGAGAGCAATTGAGC	Cassette12_7	CTAGTTAAAAAAGGAAATTCATATCCTAAGCTTAACCAGNNKTACATTAATGATAAAGGG	Cassette20_6	GCAACAAGACCCAAAGTGAGGGATCAGGAAGGGAGGNNKAACTATTACTGGACACTAGTA
Cassette5_1	TTCATCAATTATGAGGAGCTANNKGAACAGTTGAGCTCAGTGTCATCATTTGAAAGGTTT	Cassette12_8	CTAGTTAAAAAAGGAAATTCATATCCAAAGCTCAATCAGTCCNNKATTAATGATAAAGGG	Cassette20_7	GCAACAAGACCCAAAGTGAGGGACCAAGAGGGGAGGATGNNKTATTACTGGACACTAGTA
Cassette5_2	TTCATCAATTATGAGGAGCTAAGGNNKCAATTAAGCTCAGTGTCATCATTTGAAAGGTTT	Cassette13_1	CCAAAGCTTAACCAATCCTACNNKAACGACAAAGGGAAAGAAGTCCTCGTGCTGTGGGGC	Cassette20_8	GCAACAAGACCCAAAGTGAGGGACCAGGAGGGTAGGATGAACNNKTACTGGACACTAGTA
Cassette5_3	TTCATCAATTATGAGGAGCTAAGGGAGNNKTTGAGTTCAGTGTCATCATTTGAAAGGTTT	Cassette13_2	CCAAAGCTTAACCAATCCTACATCNNKGATAAGGGGAAAGAAGTCCTCGTGCTGTGGGGC	Cassette21_1	CAAGAAGGGAGAATGAACTATNNKTGGACCCTCGTAGAGCCGGGAGACAAAATAACATTC
Cassette5_4	TTCATCAATTATGAGGAGCTAAGAGAACAANNKAGTTCAGTGTCATCATTTGAAAGGTTT	Cassette13_3	CCAAAGCTTAACCAATCCTACATCAATNNKAAAGGTAAAGAAGTCCTCGTGCTGTGGGGC	Cassette21_2	CAAGAAGGGAGAATGAACTATTATNNKACCCTAGTCGAGCCGGGAGACAAAATAACATTC
Cassette5_5	TTCATCAATTATGAGGAGCTAAGAGAGCAGTTANNKTCAGTGTCATCATTTGAAAGGTTT	Cassette13_4	CCAAAGCTTAACCAATCCTACATTAACGATNNKGGTAAAGAAGTCCTCGTGCTGTGGGGC	Cassette21_3	CAAGAAGGGAGAATGAACTATTATTGGNNKCTCGTCGAACCGGGAGACAAAATAACATTC
Cassette5_6	TTCATCAATTATGAGGAGCTAAGGGAACAGTTAAGTNNKGTGTCATCATTTGAAAGGTTT	Cassette13_5	CCAAAGCTTAACCAATCCTACATTAATGACAAGNNKAAAGAAGTCCTCGTGCTGTGGGGC	Cassette21_4	CAAGAAGGGAGAATGAACTATTATTGGACCNNKGTAGAACCTGGAGACAAAATAACATTC
Cassette5_7	TTCATCAATTATGAGGAGCTAAGGGAGCAATTAAGTTCTNNKTCATCATTTGAAAGGTTT	Cassette13_6	CCAAAGCTTAACCAATCCTACATCAACGACAAGGGTNNKGAAGTCCTCGTGCTGTGGGGC	Cassette21_5	CAAGAAGGGAGAATGAACTATTACTGGACCCTANNKGAACCGGGAGACAAAATAACATTC
Cassette5_8	TTCATCAATTATGAGGAGCTAAGGGAGCAATTGAGCTCTGTGNNKTCATTTGAAAGGTTT	Cassette13_7	CCAAAGCTTAACCAATCCTACATCAACGATAAAGGTAAGNNKGTCCTCGTGCTGTGGGGC	Cassette21_6	CAAGAAGGGAGAATGAACTATTATTGGACACTAGTCNNKCCTGGAGACAAAATAACATTC
Cassette6_1	GAGCAATTGAGCTCAGTGTCANNKTTCGAGAGGTTTGAGATATTCCCCAAGACAAGTTCA	Cassette13_8	CCAAAGCTTAACCAATCCTACATCAATGATAAGGGTAAGGAANNKCTCGTGCTGTGGGGC	Cassette21_7	CAAGAAGGGAGAATGAACTATTACTGGACACTCGTCGAGNNKGGAGACAAAATAACATTC
Cassette6_2	GAGCAATTGAGCTCAGTGTCATCTNNKGAAAGATTTGAGATATTCCCCAAGACAAGTTCA	Cassette14_1	AATGATAAAGGGAAAGAAGTCNNKGTACTCTGGGGCATTCACCATCCATCTACTACTGCT	Cassette21_8	CAAGAAGGGAGAATGAACTATTATTGGACACTAGTAGAACCGNNKGACAAAATAACATTC
Cassette6_3	GAGCAATTGAGCTCAGTGTCATCTTTTNNKAGGTTCGAGATATTCCCCAAGACAAGTTCA	Cassette14_2	AATGATAAAGGGAAAGAAGTCCTTNNKCTGTGGGGTATTCACCATCCATCTACTACTGCT	Cassette1_Rprimer	TGATTCACACTCTGGATTTCC
Cassette6_4	GAGCAATTGAGCTCAGTGTCATCATTCGAANNKTTCGAGATATTCCCCAAGACAAGTTCA	Cassette14_3	AATGATAAAGGGAAAGAAGTCCTCGTANNKTGGGGTATCCACCATCCATCTACTACTGCT	Cassette2_Rprimer	GGACCATGAACTTGCTGTGGA
Cassette6_5	GAGCAATTGAGCTCAGTGTCATCATTTGAGAGANNKGAGATATTCCCCAAGACAAGTTCA	Cassette14_4	AATGATAAAGGGAAAGAAGTCCTCGTGCTCNNKGGTATTCACCATCCATCTACTACTGCT	Cassette3_Rprimer	TGAATTAGATGTTTCCACAAT
Cassette6_6	GAGCAATTGAGCTCAGTGTCATCTTTCGAGAGATTCNNKATATTCCCCAAGACAAGTTCA	Cassette14_5	AATGATAAAGGGAAAGAAGTCCTTGTGCTCTGGNNKATCCACCATCCATCTACTACTGCT	Cassette4_Rprimer	TCCTGGGTAACACGTTCCATT
Cassette6_7	GAGCAATTGAGCTCAGTGTCATCTTTTGAGAGGTTTGAANNKTTCCCCAAGACAAGTTCA	Cassette14_6	AATGATAAAGGGAAAGAAGTCCTCGTACTGTGGGGCNNKCATCATCCATCTACTACTGCT	Cassette5_Rprimer	TAGCTCCTCATAATTGATGAA
Cassette6_8	GAGCAATTGAGCTCAGTGTCATCTTTTGAAAGATTCGAAATANNKCCCAAGACAAGTTCA	Cassette14_7	AATGATAAAGGGAAAGAAGTCCTCGTACTCTGGGGTATTNNKCACCCATCTACTACTGCT	Cassette6_Rprimer	TGACACTGAGCTCAATTGCTC
Cassette7_1	TTTGAAAGGTTTGAGATATTCNNKAAAACCAGTTCATGGCCCAATCATGACTCGAACAAA	Cassette14_8	AATGATAAAGGGAAAGAAGTCCTTGTACTGTGGGGCATCCACNNKCCATCTACTACTGCT	Cassette7_Rprimer	GAATATCTCAAACCTTTCAAA
Cassette7_2	TTTGAAAGGTTTGAGATATTCCCTNNKACAAGCTCATGGCCCAATCATGACTCGAACAAA	Cassette15_1	GTGCTGTGGGGCATTCACCATNNKTCGACCACTGCTGACCAACAAAGTCTCTATCAGAAT	Cassette8_Rprimer	ATTGGGCCATGAACTTGTCTT
Cassette7_3	TTTGAAAGGTTTGAGATATTCCCTAAGNNKAGTTCTTGGCCCAATCATGACTCGAACAAA	Cassette15_2	GTGCTGTGGGGCATTCACCATCCTNNKACTACCGCTGACCAACAAAGTCTCTATCAGAAT	Cassette9_Rprimer	CGTTACACCTTTGTTCGAGTC
Cassette7_4	TTTGAAAGGTTTGAGATATTCCCCAAAACANNKTCTTGGCCCAATCATGACTCGAACAAA	Cassette15_3	GTGCTGTGGGGCATTCACCATCCTTCTNNKACTGCAGACCAACAAAGTCTCTATCAGAAT	Cassette10_Rprimer	TGCTCCAGCGTGAGGACATGC
Cassette7_5	TTTGAAAGGTTTGAGATATTCCCCAAGACCAGCNNKTGGCCCAATCATGACTCGAACAAA	Cassette15_4	GTGCTGTGGGGCATTCACCATCCATCGACTNNKGCAGACCAACAAAGTCTCTATCAGAAT	Cassette11_Rprimer	TATCAAGTTTTTGTAGAAGCT
Cassette7_6	TTTGAAAGGTTTGAGATATTCCCTAAAACCAGCTCTNNKCCCAATCATGACTCGAACAAA	Cassette15_5	GTGCTGTGGGGCATTCACCATCCATCTACCACCNNKGACCAACAAAGTCTCTATCAGAAT	Cassette12_Rprimer	TGAATTTCCTTTTTTAACTAG
Cassette7_7	TTTGAAAGGTTTGAGATATTCCCTAAGACCAGCTCTTGGNNKAACCATGACTCGAACAAA	Cassette15_6	GTGCTGTGGGGCATTCACCATCCTTCGACCACCGCANNKCAACAAAGTCTCTATCAGAAT	Cassette13_Rprimer	GTAGGATTGGTTAAGCTTTGG
Cassette7_8	TTTGAAAGGTTTGAGATATTCCCTAAGACCAGTTCATGGCCTNNKCATGACTCGAACAAA	Cassette15_7	GTGCTGTGGGGCATTCACCATCCTTCTACCACCGCTGATNNKCAAAGTCTCTATCAGAAT	Cassette14_Rprimer	GACTTCTTTCCCTTTATCATT
Cassette8_1	AAGACAAGTTCATGGCCCAATNNKGATTCTAACAAAGGTGTAACGGCAGCATGTCCTCAC	Cassette15_8	GTGCTGTGGGGCATTCACCATCCTTCTACTACCGCAGATCAANNKAGTCTCTATCAGAAT	Cassette15_Rprimer	ATGGTGAATGCCCCACAGCAC
Cassette8_2	AAGACAAGTTCATGGCCCAATCACNNKTCGAATAAAGGTGTAACGGCAGCATGTCCTCAC	Cassette16_1	TCTACTACTGCTGACCAACAANNKCTTTACCAGAATGCAGATGCATATGTTTTTGTGGGG	Cassette16_Rprimer	TTGTTGGTCAGCAGTAGTAGA
Cassette8_3	AAGACAAGTTCATGGCCCAATCATGATNNKAATAAGGGTGTAACGGCAGCATGTCCTCAC	Cassette16_2	TCTACTACTGCTGACCAACAAAGCNNKTATCAAAATGCAGATGCATATGTTTTTGTGGGG	Cassette17_Rprimer	TGCATCTGCATTCTGATAGAG
Cassette8_4	AAGACAAGTTCATGGCCCAATCACGACTCTNNKAAGGGTGTAACGGCAGCATGTCCTCAC	Cassette16_3	TCTACTACTGCTGACCAACAAAGCCTCNNKCAGAACGCAGATGCATATGTTTTTGTGGGG	Cassette18_Rprimer	TCTTGATGTCCCCACAAAAAC
Cassette8_5	AAGACAAGTTCATGGCCCAATCATGATTCGAACNNKGGGGTAACGGCAGCATGTCCTCAC	Cassette16_4	TCTACTACTGCTGACCAACAAAGTCTTTATNNKAACGCAGATGCATATGTTTTTGTGGGG	Cassette19_Rprimer	TTCCGGCTTGAACTTCTTGCT
Cassette8_6	AAGACAAGTTCATGGCCCAATCATGACTCTAATAAANNKGTAACGGCAGCATGTCCTCAC	Cassette16_5	TCTACTACTGCTGACCAACAAAGTCTCTACCAANNKGCAGATGCATATGTTTTTGTGGGG	Cassette20_Rprimer	CCTCACTTTGGGTCTTGTTGC
Cassette8_7	AAGACAAGTTCATGGCCCAATCACGATTCGAACAAGGGTNNKACGGCAGCATGTCCTCAC	Cassette16_6	TCTACTACTGCTGACCAACAAAGCCTTTACCAAAACNNKGATGCATATGTTTTTGTGGGG	Cassette21_Rprimer	ATAGTTCATTCTCCCTTCTTG

Supplementary Table 9: Cryo-EM data collection, refinement and validation statistics for NI04359_d30_245 and NI01056_d30_604.

**Map**	**Fab NI04359_d30_245 + SI06 HA**	**Fab NI01056_d30_604 + SI06 HA**
EMDB	EMD-70502	EMD-70503
PDB	9OI2	9OI3
**Data collection and processing**		
Magnification	81,000	81,000
Voltage (kV)	300	300
Electron exposure (e^-/Å^2)	57.35	57.35
Defocus range (µm)	-0.5 – 3.0	-0.5 – 3.0
Pixel size (Å)	0.529	0.529
Symmetry imposed	C1	C1
Initial particle images (no.)	1,280,356	460,651
Final particle images (no.)	256,135	286,029
Map resolution (FSC threshold 0.143; Å)	2.80	2.71
Map postprocessing	DeepEMhancer	DeepEMhancer
**Refinement**		
Model composition		
Non-hydrogen atoms	14,653	16,351
Protein residues	1,928	2,149
Root-mean-square deviations		
Bond lengths (Å)	0.005	0.005
Bond angles (°)	0.663	0.702
Validation		
MolProbity score	1.99	1.95
Clashscore	7.14	8.23
Poor rotamers (%)	2.43	1.67
Ramachadran plot		
Favored (%)	95.63	95.13
Allowed (%)	4.37	4.78
Disallowed (%)	0.00	0.10
